# Weighted Symbolic Dependence Metric (wSDM) for fMRI resting-state connectivity: A multicentric validation for frontotemporal dementia

**DOI:** 10.1038/s41598-018-29538-9

**Published:** 2018-07-25

**Authors:** Sebastian Moguilner, Adolfo M. García, Ezequiel Mikulan, Eugenia Hesse, Indira García-Cordero, Margherita Melloni, Sabrina Cervetto, Cecilia Serrano, Eduar Herrera, Pablo Reyes, Diana Matallana, Facundo Manes, Agustín Ibáñez, Lucas Sedeño

**Affiliations:** 1 Laboratory of Experimental Psychology and Neuroscience (LPEN), Institute of Cognitive and Translational Neuroscience (INCYT), INECO Foundation, Favaloro University, Buenos Aires, Argentina; 2 Fundación Escuela de Medicina Nuclear (FUESMEN) and Comisión Nacional de Energía Atómica (CNEA), Buenos Aires, Argentina; 3 Instituto Balseiro and Facultad de Ciencias Exactas y Naturales, Universidad Nacional de Cuyo (UNCuyo), Mendoza, Argentina; 4 National Scientific and Technical Research Council (CONICET), Av. Rivadavia 1917, C1033AAJ Buenos Aires, Argentina; 5 Faculty of Education, National University of Cuyo (UNCuyo), Sobremonte 74, C5500 Mendoza, Argentina; 6 Departamento de Educación Física y Salud, Instituto Superior de Educación Física, Universidad de la República, Montevideo, Uruguay; 7 Neurologia Cognitiva. Hospital Cesar Milstein., Buenos Aires, Argentina; 8 Universidad Icesi, Departamento de Estudios Psicologicos, Cali, Colombia; 9 Intellectus Memory and Cognition Center, Aging Institute, Mental Health and Psychiatry Department, Hospital Universitario San Ignacio, Pontificia Universidad Javeriana, Bogotá, Colombia; 10 Centre of Excellence in Cognition and its Disorders, Australian Research Council (ARC), Sydney, Australia; 11 Center for Social and Cognitive Neuroscience (CSCN), School of Psychology, Universidad Adolfo Ibáñez, Diagonal Las Torres, 2640 Santiago de Chile, Chile; 12 Universidad Autónoma del Caribe, Calle 90, No 46-112, C2754 Barranquilla, Colombia; 13 Instituto de Ingeniería Biomédica, Facultad de Ingeniería, Universidad de Buenos Aires, Ciudad de Buenos Aires, Argentina

## Abstract

The search for biomarkers of neurodegenerative diseases via fMRI functional connectivity (FC) research has yielded inconsistent results. Yet, most FC studies are blind to non-linear brain dynamics. To circumvent this limitation, we developed a “weighted Symbolic Dependence Metric” (wSDM) measure. Using symbolic transforms, we factor in local and global temporal features of the BOLD signal to weigh a robust copula-based dependence measure by symbolic similarity, capturing both linear and non-linear associations. We compared this measure with a linear connectivity metric (Pearson’s R) in its capacity to identify patients with behavioral variant frontotemporal dementia (bvFTD) and controls based on resting-state data. We recruited participants from two international centers with different MRI recordings to assess the consistency of our measure across heterogeneous conditions. First, a seed-analysis comparison of the salience network (a specific target of bvFTD) and the default-mode network (as a complementary control) between patients and controls showed that wSDM yields better identification of resting-state networks. Moreover, machine learning analysis revealed that wSDM yielded higher classification accuracy. These results were consistent across centers, highlighting their robustness despite heterogeneous conditions. Our findings underscore the potential of wSDM to assess fMRI-derived FC data, and to identify sensitive biomarkers in bvFTD.

## Introduction

Functional connectivity (FC) research shows that the brain is intrinsically organized via long-range networks^1–3^, whose alterations could constitute key biomarkers underlying hallmark deficits across neuropsychiatric conditions and, more particularly, neurodegenerative diseases^4–9^. Yet, the sensitivity and specificity of such disturbances remains controversial^5,6,10^. In particular, most FC studies on these diseases are based solely on linear correlation measures, such as Pearson’s correlation coefficient (R)^11–13^, which are blind to concomitant non-linear interactions in brain connectivity^14–17^. To bridge this gap, we developed a novel measure for fMRI data, inspired in a robust non-linear EEG measure called weighted Symbolic Mutual Information (wSMI)^18^, and examined whether it surpassed R in discriminating patients with behavioral variant frontotemporal dementia (bvFTD) from healthy controls, all recruited from two international centers.

The coexistence of linear and non-linear interactions in brain connectivity^16,17,19,20^ is exceedingly simplified by the linearity assumption of most FC studies on neuropsychiatric diseases^4,5,11–13^, which may be better characterized via nonlinear approaches^21^. Previous attempts in this direction, illustrated by fMRI studies based on the mutual information (MI) metric^13,22^, have failed to provide substantial new information compared to linear FC measures^14,23^, even in the comparison between healthy controls and neuropsychiatric patients^24^. This may be so because traditional MI calculations estimate probability density functions via a histogram approach, which proves inadequate for the small number of samples and low temporal resolution of fMRI data^23^.

Notwithstanding, more robust findings might be attained through wSMI^18^, a measure devised for EEG analysis which identifies non-linear coupling by transforming recorded signals into discrete sets of temporally aligned symbols, according to the time order of the neighboring data points^18,21,25^. The ensuing value is then multiplied by a given weight to correct for specific EEG artifacts, like volume conduction and dipole effects^18^. This approach has been used to assess network connectivity of socio-cognitive processes through intracranial recordings^26^ and to detect distributed EEG markers of interoception^27^ and, more crucially, neurodegeneration^28^. Moreover, discrimination between bvFTD patients and controls via neuropsychological tests improves upon inclusion of (EEG-derived) wSMI results, further highlighting the sensitivity of this measure to tap into neurodegenerative patterns^29^. Crucially, then, tapping into intrinsic fMRI connectivity with a similar approach may reveal even more robust markers of neurodegeneration.

Yet, weighted symbolic FC measures have not been applied to fMRI data, arguably because of the latter’s low temporal resolution. wSMI measures non-linear coupling through a joint probability matrix (i.e. a joint histogram)^18^. When considering long time-series, such as those of EEG signals, histograms yield accurate representations of the distributions. However, this is not the case for the sluggish signals obtained through fMRI. To overcome this limitation, here we employed non-parametric rank statistics with statistical copulas^30,31^. Copula-based dependence measures, such as Schweizer-Wolff’s^32^ and Hoeffding’s Phi-Square^33^ metrics, capture both linear and non-linear dependences, even if hidden in uniform background noise^34^. These measures resemble MI in that they are positive-definite and their value is zero if, and only if, the two measured time-series are independent, and larger values of those copula-dependent measures correspond to larger information sharing^30^. Of note, extant applications of copulas have been restricted to MEG and EEG recordings, under parametric assumptions^24^. Moreover, a full and reliable picture of brain connectivity also needs to consider that resting-state networks present transient activity during scan time^35^, and that the temporal history of the BOLD signal provides useful information for functional connectivity assessment^36^. Tapping into such intrinsic network connectivity may provide key insights on neurodegeneration^37^. Thus, to analyze the temporal history pattern of functional connectivity^36^, we also factored in the time order of neighboring data-points to produce symbols and then calculate a similarity metric between symbolic strings, so that a copula dependence measure can be weighted by symbolic similarity to produce a weighted Symbolic Dependence Metric (wSDM).

To test our approach, we targeted frontotemporal dementia, the second most common neurodegenerative disease in patients below age 65^38^. Accurate early diagnosis is difficult to achieve in this condition^38,39^, which undermines household economies and health systems while hindering the development of disease-modifying agents. Although FC measures have emerged as promising biomarkers for this and other neurodegenerative diseases^5,6,9,10^, their robustness remains inconclusive, with some bvFTD studies showing alterations of the salience network (SN) –a resting-state network encompassing the anterior cingulate cortex (ACC) alongside orbitofrontal and insular regions^5,6^–, and others showing similar disruptions in Alzheimer’s disease (AD)^6^ and other neuropsychiatric disorders^40^. Research on genetic forms of pre-symptomatic bvFTD casts additional doubts, as the SN may be altered^41^ or unaltered^42^. Furthermore, SN differences between bvFTD patients and controls, based on seed analysis, prove inconsistent across research centers^10^. This high variability and inconsistency, we surmise, might be partially related to the absence of non-linear approaches to fMRI-derived FC data in dementia research^4–7,9^.

Against this background, the present study evaluated whether the wSDM measure supersedes a linear measure (R) in the identification of resting-state networks and in its capacity to discriminate between bvFTD patients and controls across different recording centers. We collected resting-state fMRI recordings from both populations at two international clinics to assess which measure proved more consistent across centers and their heterogeneous acquisition conditions (e.g., differences in clinical diagnostic groups, fMRI equipment, and acquisition parameters)^10^. Then, using seed analysis, we compared results from wSMI and R following two steps. First, we analyzed results from healthy controls to establish which measure proved better at identifying the SN –a well-known resting-state network proposed as a specific FC alteration hallmark of bvFTD^5,6,43^, the Default Mode Network (DMN) –as a control network that is characteristically affected in other neurodegenerative diseases, such as AD^5,6,44^, but which has yielded inconsistent results in bvFTD^6^, and a primary sensory (visual) network –that is typically not affected in bvFTD^5,6^. Second, we compared both methods in terms of their capacity to (i) reveal specific SN differences between patients and controls, and (ii) classify between groups based on FC of the SN using support vector machines (SVM) and nearest neighbor (NN) algorithms. Considering that brain connectivity present both linear and nonlinear components, and that wSMI overcomes relevant limitations of other measures, we predicted that wSDM would yield more robust results than R in discriminating and classifying between patients and controls.

## Materials and Methods

### Participants

The study comprised 35 patients fulfilling revised criteria for probable bvFTD^45^ from two international clinical centers with extensive experience in neurodegeneration: the INECO Foundation, from Argentina (Country-1, with 20 controls and 25 bvFTD patients), and the San Ignacio University Hospital, from Colombia (Country-2, with 29 controls and 15 bvFTD patients). As in previous reports from both institutions^46,47^, clinical diagnosis was established by bvFTD experts (clinical details in Supplementary Information 1). Each sample was matched on gender, age, and education with healthy controls from its respective center (Table 1). All participants provided signed informed consent in accordance with the Declaration of Helsinki. The study protocol was approved by the institutional Ethics Committee of each center (i.e. the INECO Institutional Ethics Committee and the San Ignacio Hospital Ethics Committee).

**Table 1:** Demograhic details.

	Country-1				Country-2			
	HC	bvFTD	*F*-values	*p*-values	HC	bvFTD	*F*-values	*p*-values
N	20	20*	—	—	29	15	—	—
Age^(a)^	71.10 (5.16)	73.95 (5.87)	2.65	0.11	61.29 (7.16)	65.94 (7.78)	3.80	0.06
Education^a^	16.72 (3.23)	14.30 (4.57)	2.97	0.09	14.30 (5.67)	14 (4.47)	0.03	0.86
			Chi-square	*p*-values			Chi-square	*p*-values
Gender^b^	F = 12	F = 9	0.90	0.34	F = 16	F = 11	1.09	0.29
	M = 8	M = 11			M = 12	M = 4		

*Five of the 25 bvFTD patients from Country-1 were discarded after preprocessing; therefore, all reported analyses (including demographic comparisons) were performed only with the remaining 20 participants.

^a^ANOVA test. Mean (standard deviation).

^b^Chi-square test.

HC: Healthy control.

bvFTD: behavioral variant frontotemporal dementia.

### Image acquisition

Participants from both centers underwent MRI protocols including structural and resting-state sequences. Acquisition and preprocessing steps for each center are reported following the practical guide of the Organization for Human Brain Mapping (OHBM) (see Supplementary information 2 and Supplementary Table 1 for details)^48,49^.

### Preprocessing of fMRI data

Images were preprocessed using the Data Processing Assistant for Resting-State fMRI (DPARSF V.2.3) software^50^ and following previous studies^10,28,51^. Briefly, the first five volumes were discarded, and then images were slice-time corrected, aligned to the first scan of the session, corrected by nuisance regressions of the white matter and cerebrospinal fluid signals and the six head-motion parameters, normalized to the MNI space, smoothed with an 8-mm full-width half-maximum Gaussian kernel, and finally band-pass filtered (0.01–0.08 Hz). Five bvFTD patients were omitted from Country-1 because of excessive motion (>3 mm/°), yielding the final sample of 20 patients (further details in Supplementary Information 3 and Supplementary Table 2).

### Seed analysis

Consistency across controls and differences between groups across countries were explored in the SN and the DMN. We also included a visual network as a complementary control resting-state network that is not typically affected in bvFTD^5,6^. Seed maps were obtained by using R and wSDM. We placed two bilateral seeds for each network; one pair was located on the posterior cingulate cortex (PCC), a key node of the DMN^52^, other on the dorsal anterior cingulate cortex (dACC), a main hub of the SN^9^, and other on the higher visual cortex^53^. Seed maps were obtained by using R and wSDM (see details in Supplementary Information 4).

#### Pearson correlation coefficient

To calculate R, we employed the standard linear correlation function included in MATLAB. We discarded negative correlations –setting them to zero^10,51,54,55^, because their interpretation is controversial in resting-state studies^3^.

#### wSDM

Dependence measures such as MI and wSMI tap into non-linear dependencies yet their application in fMRI studies is limited because of their low temporal resolution^56^. One way to tackle this problem is to use rank statistics, such as in the case of dependency measures based on statistical copulas^30^. In this way, consider the MI of two random variables ${\rm{x}},{\rm{y}}$:

1 $$MI(x,y)=\iint f({u}^{1},\,{u}^{2})log[\frac{f({u}^{1},{u}^{2})}{{f}_{1}({u}^{1}){f}_{2}({u}^{2})}]d{u}^{1}d{u}^{2}$$

where ${\rm{f}}$ is the joint probability density function (PDF), and ${{\rm{f}}}_{1}$, ${{\rm{f}}}_{2}$ its marginal PDF. ${\rm{MI}}$ is non-negative and it is zero if, and only if, the variables are independent^57^. We can also define MI as a function of the copula, as shown in^58^:

2 $$MI(x,y)=\iint c({u}^{1},{u}^{2})log[c({u}^{1},{u}^{2})]d{u}^{1}d{u}^{2}$$

However, this double integral may not have analytical solution.

Let C be the copula function of the random variables $({\rm{x}},{\rm{y}})$ defined on a unit square. According to Sklar’s theorem^59^, there exists a unique copula C that links the joint distribution ${\rm{f}}$ and the marginals ${{\rm{f}}}_{1}$, ${{\rm{f}}}_{2}$:

3 $${\rm{f}}({\rm{x}},{\rm{y}})={\rm{C}}({{\rm{f}}}_{1}({\rm{x}}),{{\rm{f}}}_{2}({\rm{y}}))$$

Using the result that the variables ${\rm{x}},{\rm{y}}$ are independent if and only if the copula C equals the product copula П defined as the product of their marginal distribution functions^30^, the independence of the variables can be measured by a normalized ${{\rm{L}}}^{{\rm{P}}}$ distance of C and П:

4 $${({{\rm{h}}}_{{\rm{p}}}{\iint }_{{[0,1]}^{2}}|{\rm{C}}({{\rm{u}}}^{1},{{\rm{u}}}^{2})-\prod ({{\rm{u}}}^{1},{{\rm{u}}}^{2})|{{\rm{du}}}^{1}{{\rm{du}}}^{2})}^{\frac{1}{{\rm{p}}}},$$

where 1 ≤ p ≤ ∞ and ${{\rm{h}}}_{{\rm{p}}}$ is a normalization constant.

For p = 1 and p = ∞ we obtain Schweitzer-Wolff’s σ and κ, respectively:^32^

5 $${{\rm{ISW}}}_{{\rm{\sigma }}}=12{\iint }_{{[0,1]}^{2}}|{\rm{C}}({{\rm{u}}}^{1},{{\rm{u}}}^{2})-\prod ({{\rm{u}}}^{1},{{\rm{u}}}^{2})|{{\rm{du}}}^{1}{{\rm{du}}}^{2}$$

and

6 $${\mathrm{ISW}}_{{\rm{\kappa }}}=4\,{\rm{\sup }}|{\rm{C}}({{\rm{u}}}^{1},{{\rm{u}}}^{2})-\prod ({{\rm{u}}}^{1},{{\rm{u}}}^{2})|$$

where $\sup \,\,$is the supremum under the unit square.

For p = 2, we have Hoeffding’s phi-square (${\rm{I}}{{\rm{\varphi }}}^{2})$^33^,

7 $$\,{\rm{I}}{{\rm{\varphi }}}^{2}=90{\iint }_{{[0,1]}^{2}}|{\rm{C}}({{\rm{u}}}^{1},{{\rm{u}}}^{2})-\prod ({{\rm{u}}}^{1},{{\rm{u}}}^{2})|{{\rm{du}}}^{1}{{\rm{du}}}^{2}$$

whose empirical estimation can be analytically computed^60^. We targeted this dependence measure (${\rm{I}}{{\rm{\varphi }}}^{2}$) because it can be accurately estimated. To calculate it, we employed the Information Theoretical Estimators (ITE) Toolbox^61^.

The dependency measure based on statistical copulas^31,34,56^ enables us to estimate MI -like dependence measures despite the low temporal resolution of the fMRI data. However, another limitation of the MI measure (also present in R) is that it overlooks the temporal order of the data points in the time series. Hence, it only provides a static description of FC, thus proving blind to key aspects of neural connectivity^14,62,63^. However, FC can be also studied by considering the information contained within the BOLD signal’s temporal history pattern^36^. To account for local increases and decreases of the signal, the neighboring values of each time-series data points can be compared to produce symbols via a symbolic transform^21^. Then, we can measure the similarity between two symbol strings considering their respective arrangements to account for time-dependent, global signal modulations.

Let us define a symbolic weight *sw* which is function of the similarity of $\hat{{\rm{X}}},\hat{{\rm{Y}}}\,$(i.e., the symbolic transformation of the ${\rm{x}},{\rm{y}}$ timeseries). By multiplying the copula-based measure ${\rm{I}}({\rm{x}},{\rm{y}})$ we obtain the formula for the wSDM:

8 $${\rm{wSDM}}={\rm{sw}}(\hat{{\rm{X}}},\hat{{\rm{Y}}}).{\rm{I}}({\rm{x}},{\rm{y}})$$

The symbolic weights, which range from 0 (i.e., minimal similarity) to 1 (i.e., maximal similarity), were calculated using the Hamming distance^64^ between the obtained symbolic strings. As this index only measures the minimum number of substitutions needed to modify one string to match the other, it is suitable for strings of the same length (e.g., two transformed fMRI time series). Moreover, the Hamming distance is fast to compute in large datasets, such as those comprised of fMRI volumes.

We applied a symbolic transform considering the adjacent neighboring values of each time-series and an output of two possible symbols (i.e., a symbol for the local increase of the signal, and another symbol for the local decrease of the signal). To better understand all the procedures involved in the calculations of the wSDM measure, we have included a flowchart (Supplementary Fig. 1). To evaluate the contribution of the weighted symbols for the dependency measure, we compared our classification results based on the wSDM measures with the ones estimated without applying any symbolic transformation (these results are depicted in Supplementary Table 3 and Supplementary Table 4).

Finally, before we applied the wSDM to the neuroimaging data, we tested it with a simple artificial model to corroborate that it was able to capture non-linear associations (Supplementary Information 5).

### Thresholding

We applied a proportional threshold to the association values instead of setting it to a fixed value (e.g. R = 0.3) which may bias our results, given that R and wSDM have different units and voxel distributions. By employing different proportional thresholds, we assessed machine learning classification accuracy under different number of features (i.e. number of voxels). To avoid over-fitting when optimizing the threshold hyperparameter, we performed a nested cross-validation using one test set to obtain the optimum threshold and a different one to obtain unbiased accuracy rates^65^. Our main results are based on the 30^th^ percentile, which yielded most of the maximum classification accuracy rates on our machine learning analysis, relative to other thresholds (see section 3.3 and Supplementary information 6 for further details). Yet, to show that the seed-based analysis in the control sample resembles previous studies^66–69^ –in which no thresholding approach was applied–, we also report seed results of the DMN and SN in this group without thresholding for both centers (Supplementary Fig. 3 and 4).

### Statistical procedures and analysis

To assess the resting-state networks in the control sample of each dataset, we employed a one-sample *t*-test to display FC of the DMN and the SN (FWE-corrected, *p* = 0.05 at the voxel level, extent threshold = 30 voxels^70,71^). To evaluate the consistency of these results, we employed a conjunction analysis by overlapping the significant areas across centers (FWE-corrected, *p* = 0.05, extent threshold = 30 voxels)^10^. Then, connectivity differences between controls and bvFTD patients were calculated via a two-sample *t*-test (*p* < 0.001, extent threshold = 30 voxels)^10,51^. We tested the hypothesis that patients would exhibit hypo-connectivity compared to controls [bvFTD < healthy control] given that it is the most consistently finding about the SN in bvFTD^6^. We applied the same contrast for the DMN: although a few reports have shown increased connectivity of this network in bvFTD^72,73^, assessments of hypo-connectivity have more consistently revealed null or reduced differences compared to the SN^42,72–74^. These analyses were run on SPM 12^75^.

To further assess the consistency of seed-analysis findings across countries, we implemented a reproducibility metric used in previous reports^4,51^ (details in Supplementary information 7).

Finally, to identify the most robust association method to discriminate bvFTD patients from controls, we employed both a linear and a non-linear class boundary machine learning classifiers: the SVMs and k-nearest neighbor (kNN) algorithms^76^ (see details Supplementary information 8).

### Data availability

The datasets generated during and/or analysed during the current study are available from the corresponding author on reasonable request.

## Results

### Consistency of resting-state networks in controls

**Figure 1:**
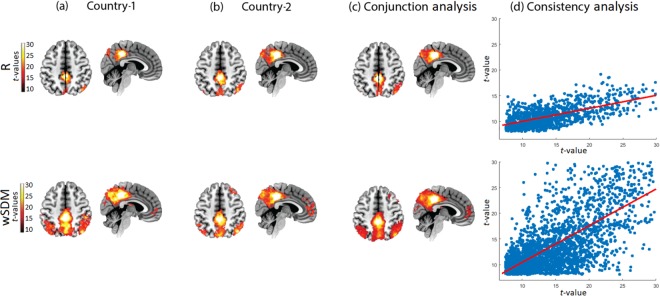
Default mode network (DMN). **(a**,**b)** Seed analysis results of the DMN using R and wSDM at the 30^th^ percentile threshold, for Country-1 and Country-2 (FWE-corrected, *p* = 0.05 on the voxel level, extent threshold = 30) (axial plane z = 48, sagittal plane x = 4). **(c)** Cluster overlap between Country-1 and Country-2 (FWE-corrected, *p* = 0.05, extent threshold = 30 voxels). **(d)** Consistency analysis based on a voxel-wise correlation analysis between the maps (*T*-values) of both countries. All brain images are presented according to neurological convention.

Regarding the DMN, both methods (R and wSDM) evidenced the main posterior regions of this network (i.e., PCC and the angular gyrus); yet, only the latter reproduced the network’s anterior section (mPFC) (Fig. 1a,b and Supplementary Table 5). This pattern of results for each method was consistent across countries, as shown by the conjunction analysis (Fig. 1c and Supplementary Table 5). However, the reproducibility metric based on the voxel-wise correlation analysis between the results of each dataset showed higher consistency values for wSDM (Rho = 0.71, *p* < 0.001) than for R (Rho = 0.25, *p* < 0.001) (Fig. 1d). High correlation values (Rho > 0.2, *p* < 0.001) indicate a large consistency of results between datasets, while low values (Rho < 0.2, *p* > 0.05) suggest that differences between samples were not consistent across countries^4^. In addition, the slope analysis of the reproducibility metric between measures showed that wSDM yielded significantly different correlation values compared to R (*t*-value = 31.11, p < 0.001). The same pattern was yielded by the analysis of the visual network, with the wSDM presenting higher consistency values across centers (see Supplementary Fig. 8 and Supplementary Table 5).

**Figure 2:**
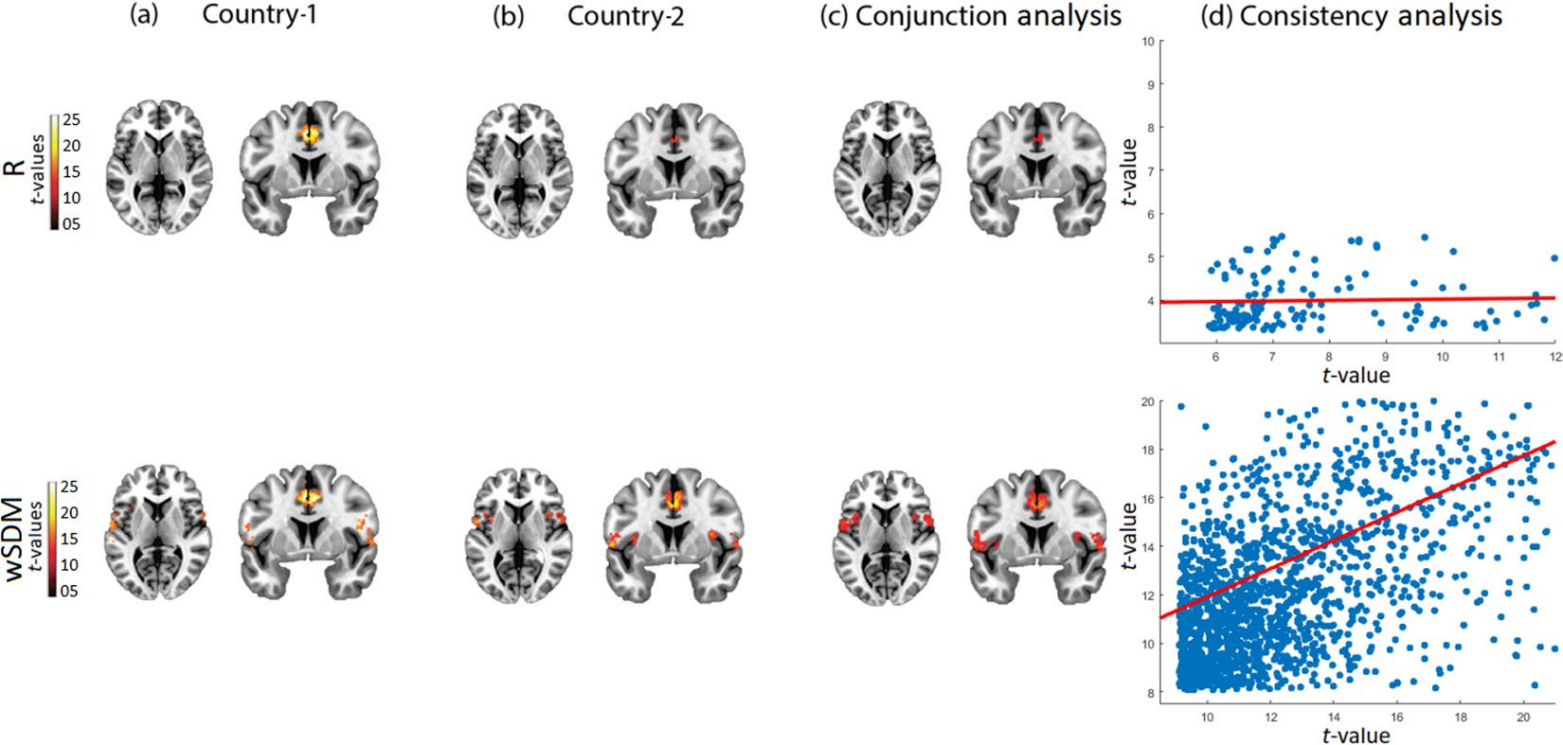
Salience network (SN). **(a**,**b)** Seed analysis results of the SN using R and wSDM at the 30^th^ percentile threshold, for Country-1 and Country-2. (FWE-corrected, *p* = 0.05 on the voxel level, extent threshold = 30) (axial plane z = 3, coronal plane y = 6). **(c)** Cluster overlap between Country-1 and Country-2 (FWE-corrected, *p* = 0.05, extent threshold = 30 voxels). **(d)** Consistency analysis based on a voxel-wise correlation analysis between the maps (T-values) of both countries. The scale of the axis is not the same between methods given the differences in T-values (data dispersion from the R method would not be well illustrated if identical scales were used for each metric). All brain images are presented according to neurological convention.

As regards the SN, both R and wSDM reproduced the main anterior regions of this network (ACC); yet, only the latter included the insula and the fronto-temporal operculum (Fig. 2a,b and Supplementary Table 5). This pattern of results for each method was consistent across countries as shown by the conjunction analysis (Fig. 2c and Supplementary Table 5). However, the reproducibility metric based on the voxel-wise correlation analysis (Fig. 2d) between the results of each dataset showed higher consistency values for wSDM (Rho = 0.58, *p* < 0.001) than for R (Rho = 0.013, *p* = 0.54). Moreover, the non-linear measure presented a significantly different slope of association compared to the linear one (*t*-value = 21.58, *p* < 0.001).

Finally, we also tested the consistency of the extension of the seed-based maps at the individual level, for each method. We found that, relative to R, wSDM presented a more significant homogeneous extension of the DMN and the SN across healthy controls in both centers. As expected, the same was true for patients, but only for the DMN; instead, the SN presented more heterogeneous results, which is consistent with the alteration of this network in bvFTD^5,6^ (for further details, see Supplementary Information 9).

### Functional connectivity differences between bvFTD and healthy controls

**Figure 3:**
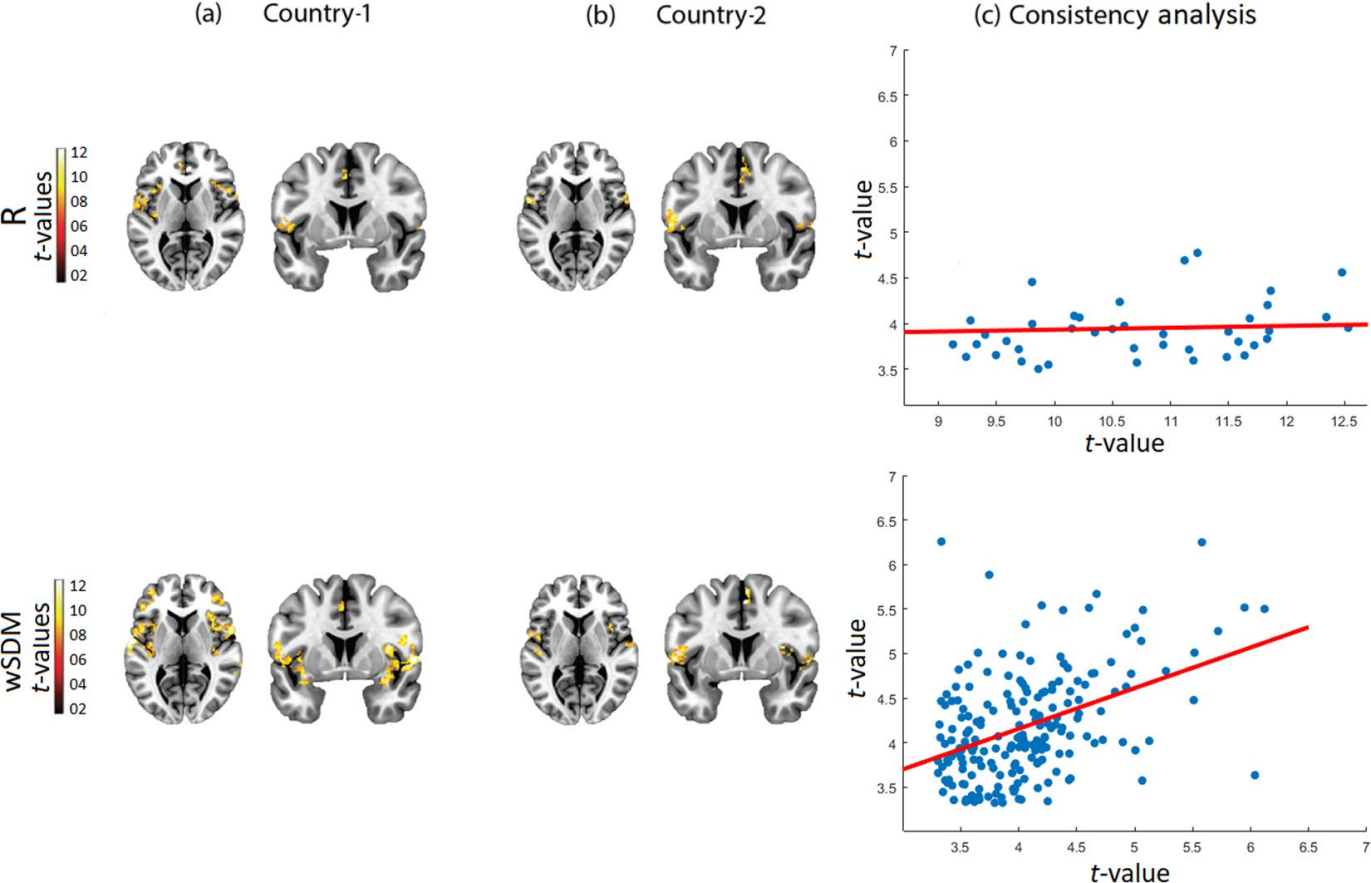
SN bvFTD vs. healthy controls. **(a**,**b)** Seed connectivity maps (axial plane z = 3, coronal plane y = 6) comparing HC > bvFTD through a two-sample *t*-test in the two centers showed a very consistent engagement of the insular cortex and the ACC, two main hubs of the SN. The connectivity volumes have been previously thresholded at the 30^th^ percentile threshold, while the SPM threshold was set to *p* = 0.001, extent threshold = 30 voxels. **(c)** Consistency analysis based on a voxel-wise correlation analysis between the maps (T-values) of both countries. The scale of the axis is not the same between methods given the differences in T-values (data dispersion from the R method would not be well illustrated if identical scales were used for each metric). All brain images are presented according to neurological convention.

To evaluate which method was the more powerful to detect connectivity differences between controls and bvFTD patients across centers, we employed two-sample *t*-tests. Figure 3 shows the connectivity differences after applying the healthy controls > bvFTD contrast for the SN (see also and Supplementary Table 5).

Results from the R method showed a similar pattern of reduced connectivity of the SN in bvFTD for both datasets (Fig. 3: bilateral insular cortex, left fronto-temporal operculum, and left ACC for Country-1; left insular cortex, left fronto-temporal operculum, and right ACC for Country-2). With the wSDM method we also found differences in the main hubs of the SN between groups, but these were larger and covered a greater extension than the ones from R (Fig. 3: bilateral insular cortex and fronto-temporal operculum, and left ACC for Country-1; bilateral insular cortex and fronto-temporal operculum, and right ACC for Country-2). The reproducibility metric based on the voxel-wise correlation analysis between the results of each dataset (as performed above for the analysis of networks only in the control sample) showed higher consistency values for wSDM (Rho = 0.45, *p* < 0.001) than for R (Rho = 0.04, *p* = 0.06). This difference was further supported by the significant higher slope of the non-linear measure compared to the linear one (*t*-value = 5.68, *p* < 0.001).

**Figure 4:**
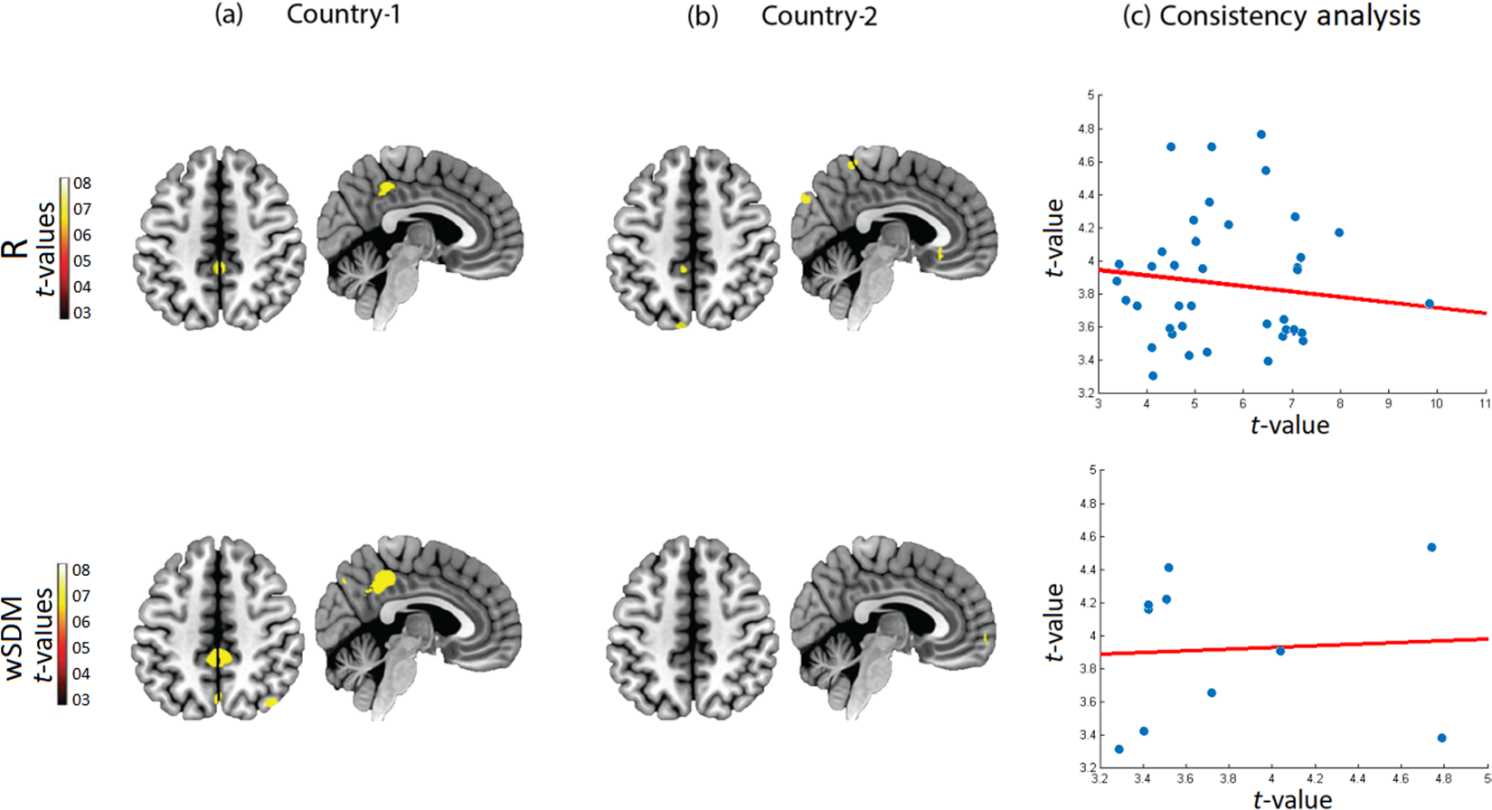
DMN bvFTD vs. healthy controls. **(a**,**b)** Seed connectivity maps (axial plane z = 48, sagittal plane 6) comparing (bvFTD < HC) through a two-sample *t*-test in the two centers showed a very consistent engagement of the insular cortex and the ACC, two main hubs of the SN. The connectivity volumes have been previously thresholded at the 30^th^ percentile threshold, while the SPM threshold was set to *p* = 0.001, extent threshold = 30 voxels. **(c)** Consistency analysis based on a voxel-wise correlation analysis between the maps (T-values) of both countries. The scale of the axis is not the same between methods given the differences in T-values (data dispersion from the R method would not be well illustrated if identical scales were used for each metric). All brain images are presented according to neurological convention.

To evaluate the specificity of the SN results, we also compared the DMN and the visual network between patients and controls across centers. None of the methods showed consistent differences between samples, and both presented low reproducibility values across centers (see Fig. 4 for the DMN, and Supplementary Fig. 9 for the visual network). As expected, this suggests that SN alterations may represent a specific (and more consistent than DMN) hallmark for bvFTD.

### Machine learning analysis

**Figure 5:**
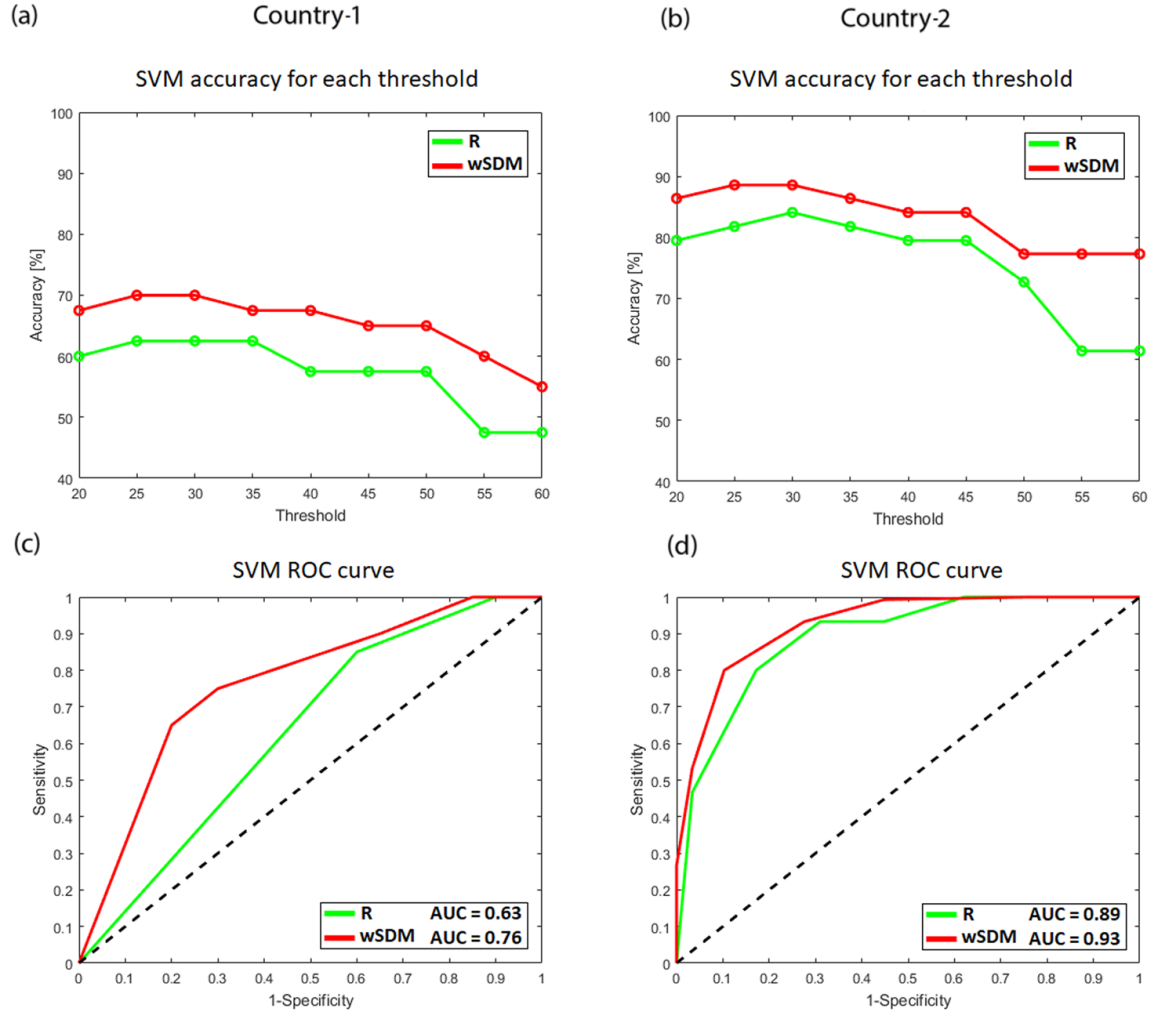
Accuracy and ROC curves for the SVM classifier. **(a**,**b)** Classification accuracy for Country-1 and Country-2 while varying the percentile threshold, for R and wSDM (see Supplementary Table 3 for details). (**c**,**d**) ROC curves (AUC significance, p < 0.01) [Sensitivity (TPR) vs. 1-Specificity (FPR)] graph for Country-1 and Country-2, for wSDM and R, considering the optimal threshold. The area under the curve (AUC) measures the performance of the classifier across different points of the ROC space. The dashed black line represents random guess (i.e., AUC = 50).

To evaluate which connectivity method was more accurate to classify bvFTD patients from controls, first we employed the same SVM classifier (i.e., with the same kernel and the same parameters) for both measures and countries across different thresholds. In order to determine the threshold that provides maximum classification accuracy, the latter was calculated while incrementing the percentile threshold to find the maximum accuracy (Fig. 5a,b). This classifier showed that for both countries, the wSDM measure is more accurate than the R measure under all the percentile thresholds, with maximum accuracies emerging between the 25^th^ and the 30^th^ percentile. At the optimum threshold (30^th^), wSDM achieved an accuracy rate of 70% for Country-1 and of 88.6% for Country-2, while R achieved an accuracy rate of 62.5% for Country-1 and of 84.1% for Country-2. To further analyze the classification scores, we plotted the ROC curves for both countries and both measures under their optimum threshold for each measure (Fig. 5c,d). We can see that higher AUC values were obtained for wSDM relative to R, for both countries (wSDM achieved an AUC of 0.76 for Country-1 and of 0.93 for Country-2, while R achieved an AUC of 0.63 for Country-1 and of 0.89 for Country-2).

The SVM classifier, showed statistically significant differences indicating higher classification rates for wSDM than for R across centers (Country-1: score = 78, *p* < 0.001; and Country-2: score = 63.5, *p* = 0.04). In addition, we also compared the performance of an unweighted symbolic dependence measure (DM) relative to R and wSDM in the two countries, to evaluate the effects of adding the symbolic weights. We found that, although DM yielded better results than R, wSDM outperformed both measures (mean classification rates: for R, 57.22% for Country-1, 75.74% for Country-2; for DM, 63.88% for Country-1, 82.32% for Country-2; and for wSDM, 65.83% for Country-1, 83.58% for Country-2) (see Supplementary Fig. 10 and Supplementary Fig. 11).

**Figure 6:**
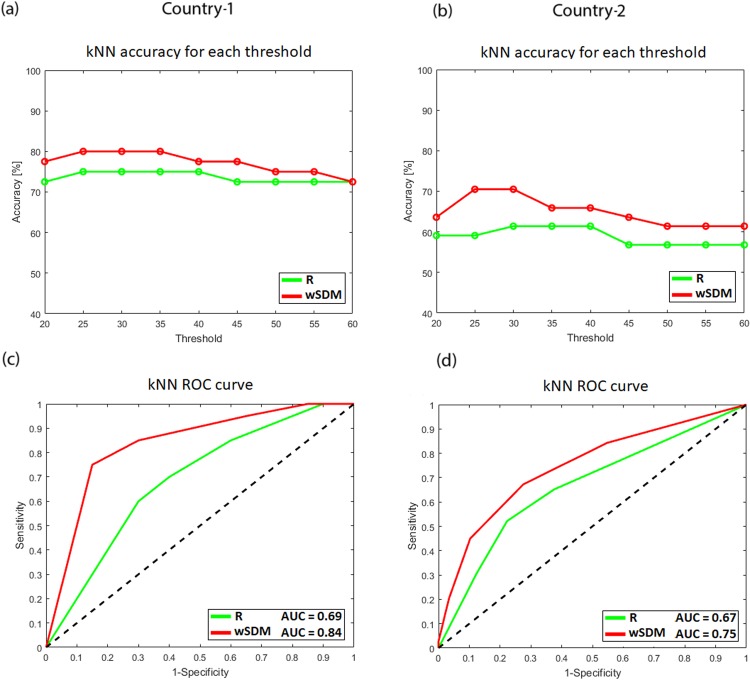
Accuracy and ROC curves for the KNN classifier. **(a**,**b)** Classification accuracy for Country-1 and Country-2 while varying the percentile threshold (i.e., number of features), for R and wSDM (see Supplementary Table 4 for details). **(c**,**d)** ROC curves (AUC significance, *p* < 0.01) [Sensitivity (TPR) vs. 1-Specificity (FPR)] graph for Country-1 and Country-2, for wSDM and R, considering the optimal threshold. The area under the curve (AUC) measures the performance of the classifier across different points of the ROC space. The dashed black line represents random guess (i.e., AUC = 50).

We repeated the same analysis using the kNN classifier to test both measures with a non-linear class boundary. We employed this classifier under the same settings and parameters to test for classification accuracy for R and wSDM. As with SVM, we searched the optimal threshold (Fig. 6a,b). For both countries, the wSDM measure was more accurate than R nearly in all percentile thresholds, yielding maximum accuracies between the 25^th^ and the 35^th^ percentile. At its optimal threshold, wSDM achieved an accuracy rate of 80% for Country-1 and of 70.5% for Country-2, while R achieved an accuracy rate of 75% for Country-1 and of 61.4% for Country-2. To further analyze classification scores, we also plotted the ROC curves for both countries and both measures under their optimal threshold for each measure (Fig. 6c,d): higher AUC were obtained for wSDM when compared to R, for both countries (wSDM achieved an AUC of 0.84 for Country-1 and of 0.75 for Country-2, while R achieved an AUC of 0.69 for Country-1 and of 0.67 for Country-2).

In the kNN classifier, we also found significant differences between methods indicating higher classification rates for wSDM compared to R across centers (Country-1: score = 73, p < 0.001; and Country-2: score = 60.5, p = 0.007). In addition, we repeated the comparison between these measures and DM but with kNN classification values, and we found that wSDM still outperformed both DM and R across countries (mean classification rates: for R, 73.61% for Country-1, 60.37% for Country-2; for DM, 76.11% for Country-1, 58.58% for Country-2; and for wSDM, 77.22% for Country-1, 64.91% for Country-2) (see Supplementary Fig. 12 and Supplementary Fig. 13). Moreover, as a recent study highlighted that partial correlations tend to outperform R in the discrimination between AD patients and healthy controls^77^, we tested the wSDM against this coupling measure. Given that estimating partial correlation for voxel-wise analysis is extremely demanding in computational terms^78^, we followed Vos and colleagues^77^ procedures, and performed the analysis parcellating the brain into large homogeneous regions. To this end, we relied on the Automated Anatomical Labeling (AAL) atlas^79^ –one of the most broadly used atlases in network studies of neurodegenerative diseases^34,43,80–89^ to select the regions related to the SN –as in a previous work of our group^51^. Partial correlations and wSDM within the regions of the SN were tested with the same classification methods applied for our seed-based analysis. As expected, in both countries, we found that partial correlations surpassed R but not wSDM classification rates (see Supplementary information 10). Moreover, the wSDM results with this approach were lower than the ones obtained with the seed-based analysis reported in the main manuscript (65% to 67% in the latter, compared to 70% to 88% in the seed-based analysis). This finding further supports the relevance of fine-grained FC data to discriminate patients from healthy controls.

Finally, to test the specificity of the SN for bvFTD, we repeated the same analysis using the DMN and the visual network. In the former, SVM and kNN yielded low mean classification rates (near chance) [SVM for R (50.55% Country-1, 58.61% Country-2) and for wSDM (63.88% Country-1, 69.46% Country-2), and kNN for R (51.38% Country-1, 48.62% Country-2) and for wSDM (52.22% Country-1, 44.71% Country-2)] (Supplementary Tables 6 and 7). Low classification results were also found for the visual network with both methods and in both countries (Supplementary Table 8). In sum, these results highlight the specificity of the SN alterations detected in bvFTD patients.

## Discussion

This multicenter study aimed to assess the robustness of the wSDM approach as a novel non-linear association method for fMRI resting-state analysis of neurodegenerative brain networks. Relative to a widely used linear metric (namely, R), the wSDM measure proved superior at identifying resting-state networks, revealing specific differences between bvFTD patients and controls, and classifying groups via machine learning analysis. Notably, such results were consistent despite the heterogeneous acquisition and sociocultural contexts of the centers involved. These findings highlight the potential of wSDM to assess FC based on fMRI data, and, more particularly, to identify sensitive biomarkers in bvFTD (and potentially other neurodegenerative diseases).

### Linear vs. non-linear methods

Our novel wSDM measure outperformed R in the detection and analysis of resting-state networks. First, based on a seed analysis, the wSDM successfully identified two well-characterized resting-state networks in healthy subjects, namely: the DMN and the SN^38,44,52,90^. While this was also possible with R, the wSDM approach evinced higher consistency across centers with heterogeneous acquisition conditions. This result suggests that a measure which captures both linear and non-linear connectivity, such as wSDM, could prove more reliable and resistant against the variability of data with diverse characteristics and from different sources. The same was true in the comparison between patients and controls. Relative to R, wSDM revealed more consistent alterations of the SN (a hallmark disturbance in bvFTD^5,6,43^) across countries. Yet, both methods presented inconsistent results across centers when the DMN was analyzed. This pattern of results was expected given that previous studies have reported null or reduced hypo-connectivity differences within this network in bvFTD^42,72–74^. Moreover, the visual network also failed to show consistent differences between samples. Taken together, our findings reinforce the specificity of SN disturbances as a potential hallmark of bvFTD. Additional support for the robustness of wSDM came from the machine learning analysis. Two different approaches (based on linear or non-linear boundaries) showed that this measure achieved significantly higher classification rates than R in both countries. As before, this result was based on the FC of the SN, while the DMN and the visual network presented classification rates near chance. Such findings further evince the relevance of a measure that combines both linear and non-linear properties for obtaining consistent results in heterogeneous contexts.

Although brain function presents non-linear properties^15,17,91,92^, the implementation of non-linear FC methods has yielded inconsistent results as compared to linear ones. Non-linear measures have been found to provide useful information to discriminate patients (e.g., in consciousness impairments and schizophrenia) and different experimental conditions^93–95^. For example, an EEG study found that MI measures superseded Pearson, Spearman, and Kendall correlations in discriminating between brain states^93^. In addition, MI also outperformed the traditional general lineal model in establishing significant differences between specific stimulus types via fMRI correlates^94^. However, while non-linear brain connectivity analysis on schizophrenia using a measure based on MI found disease-specific pathological non-linear connections, this approach did not prove superior to R in classifying between patients and healthy controls^95^. Moreover, another fMRI study on schizophrenia found higher differences in FC between patients and controls via linear correlation than through MI^23^. In addition, other non-linear approaches to EEG research, such as wSMI, have been observed to surpass traditional FC measures like Phase Locking Value and Phase Lag Index in detecting patients with consciousness impairments^18^. However, these robust features are not inherent to *any* non-linear method, especially when considering fMRI data. Indeed, a resting-state fMRI study showed that, while non-gaussianity was present in the FC signal, the portion of information neglected by linear correlation amounted to only 5% compared to MI^13^. These findings cast doubts on the practical relevance of standard non-linear methods in fMRI. However, the specific non-linear measures applied so far in the field are undermined by the intrinsic low time-resolution of the technique, which hinders a reliable estimation of MI by using a standard histogram approach. In addition, those measures do not capture the temporal dimension of the signal, providing a static representation of FC which may ignore relevant time features of the BOLD signal.

Compared to FC analyses in the above fMRI studies, the wSDM presents two methodological advantages that might explain our positive results. First, its symbolic approach allows to partially capturing the temporal history information of the BOLD signal for estimating associations between regions. While most connectivity measures (linear or non-linear) are blind to this temporal dimension of the signal^36^, the wSDM measure contemplates the time order of neighboring data-points to produce symbols, and allows characterizing FC both locally (within symbols) and globally (across strings) based on the temporal history pattern of the BOLD signal. Moreover, by using weights based on symbolic similarity, associations that are incoherent in time are yield near-zero values, thus reducing FC false positives and augmenting the robustness of detected patterns. Previous studies have shown the relevance of considering the temporal dimension of FC^12,14,36^. For example, it has been essential for discriminating between patients with consciousness impairments via EEG signals^18^. Moreover, in fMRI, it improves diagnostic power in Alzheimer’s disease^96^ and allows for the detection of transient dysconnectivity of resting-state networks in schizophrenia patients^97^. Second, to overcome the limitation of the low temporal resolution of fMRI data, we applied a copula-based dependence measure, namely the Hoeffding Phi-Square^60^, which is based on copula functions that link the probability of joint and marginal signal distributions to measure dependence. Instead of approximating the distributions via a histogram to calculate MI, which may lead to inconsistent results, the copula-based approach is resistant to the effect of outliers because it is based on rank statistics^31^ and, hence, it might be more robust to estimate common features across heterogeneous data, as in the case of our study.

In sum, our report supports the sensitivity of the wSDM measure and shows that weighted MI metrics could be particularly sensitive to analyze FC from fMRI data. The consistency of our findings despite heterogeneity in acquisition methods and socio-cultural features of the data samples highlights the potential of wSDM as a robust metric for establishing widely applicable biomarkers.

### Relevance for neurodegeneration studies

Like other neurodegenerative conditions, FTD impacts household economies and health systems worldwide^98–101^. Finding robust and effective biomarkers for its early detection is thus crucial for the implementation of new re-habilitation and intervention methods to alleviate the impact of these conditions^102,103^. FC has been proposed as a potential biomarker of neurodegenerative diseases^4,6,104^, with recent studies on bvFTD focusing on SN connectivity^41,42^. However, so far linear measures have yielded inconsistent results, concluding that this network could be either altered or preserved in symptomatic and pre-symptomatic stages^41,42^. Moreover, the specificity of SN alterations also proves controversial, given that other resting-state networks (e.g., the DMN) can be affected in bvFTD^73,105^, whereas the SN can also be altered in other diseases^106,107^. In addition, a previous study based on linear correlation measures, showed that SN differences between controls and patients were not consistent across different recordings’ centers^10^.

Importantly, all these studies were based on linear correlation indexes. Thus, in light of our present findings, we propose that a dependence measure such as wSDM could potentially circumvent the inconsistency of extant FC results. Relatively few studies have employed non-linear connectivity methods to tap into pathology-specific FC alterations in neurodegeneration. Synchronization likelihood (SL)^108^, has been used to show abnormalities on long-range networks in AD patients with EEG^109^ and MEG^110^. As mentioned before, in dementia research, the wSMI approach was applied only in one previous EEG studies, in which it proved to be a robust biomarker for bvFTD^29^. This dependency measure has never been used to analyze FC in dementia patients given the methodological constraints of the fMRI signal (low time/frequency resolution) for its application. Here, we circumvent this issue by employing a copula-based approach to estimate FC based on wSDM. In addition, we showed that this measure was, compared to a linear method (R), more consistent across centers in the identification of resting-state networks, and in the discrimination and classification of patients from healthy participants.

In this way, our findings proved to be robust against acquisition heterogeneity. On the one hand, the use of statistical copulas based on non-parametric rank statistics are more resilient to outliers than parametric measures such as R, hence providing consistent results even over MR acquisitions with different signal-to-noise ratio. On the other, the symbolic weights capture transient brain activity that conventional methods, such as R and MI, may disregard as noise. Moreover, a static FC measure provides an averaged connectivity pattern across acquisition time, which may undermine FC sensitivity. By capturing these key properties of FC despite the heterogeneity of the samples and fMRI parameters, wSDM may afford sensitive biomarkers for neurodegenerative diseases across clinical centers^111,112^.

In sum, we found that the wSDM measure outperformed linear measures across centers in the discrimination and classification of patients based on the SN, and that these results were specific for this network (given an absence of consistent differences in the DMN and the visual network). Considering the underemployment of nonlinear measures to study neurodegenerative diseases in fMRI, and the variability of the results reported in previous research, our findings highlights the potential value of wSDM as a novel biomarker measure for neurodegenerative disease, although further research is needed.

### Limitations and future studies

A major limitation of this study is the moderate sample size from each center. However, similar works have reported valid, replicable results with similar or smaller samples^44,73^, and our findings prove consistent across centers and relative to previous studies^5,43^. Nevertheless, future research should test the robustness of this measure compared to linear approaches in at least two critical scenarios. First, replication studies should be conducted with larger samples, to establish whether reduced variability related to increased data availability reduces the differences between linear and non-linear methods. Second, multiple single-case analyses would be highly informative to establish the potential of our metric for diagnostic and follow-up purpose in clinical settings^113^.

Although several different regions and coordinates can be selected for seed-based analysis, we have used areas that are targets of each of the evaluated networks, namely, the SN^90^, the DMN^52^, and the visual network^53^. Moreover, we showed that the wSDM outperformed R in the characterization of all of them, which highlights the potential generalization of our non-linear method to network research beyond the selection of specific areas and coordinates for a given seed.

Looking forward, the sensitivity and specificity of wSDM should be tested in future studies including a contrastive pathological group. Also, future research in this line should also include clinical data to analyze potential associations between FC and the patients’ cognitive decline, as done elsewhere in the literature^27,29,51^.

## Conclusion

Resting state FC from fMRI data represents a promising tool for the development of early biomarkers for neurodegenerative disorders. However, despite that brain connectivity presents both linear and non-linear components, most dementia research in fMRI is based on linear dependency measures. This oversimplifying approach might explain the controversy results regarding the sensitivity and specificity of FC on these diseases. Here, we found that a novel non-linear connectivity measure consistently outperformed a linear one in the discrimination and classification of bvFTD patients from healthy participants across different centers. Our findings suggest that the wSDM might be robust against acquisition parameter heterogeneity and, hence, it has a potential value as a biomarker for neurodegenerative diseases.

## Electronic supplementary material

Supplementary Information
