# Supplementary material for: Weighted Symbolic Dependence Metric (wSDM) for fMRI resting-state connectivity: A multicentric validation for frontotemporal dementia
Source: Sci Rep. 2018 Jul 25;8:11181. doi: 10.1038/s41598-018-29538-9 (PMC6060104; doi:10.1038/s41598-018-29538-9)
Supplement: Supplementary file 1 — Supplementary Information [file 41598_2018_29538_MOESM1_ESM.docx]

**Weighted Symbolic Dependence Metric (wSDM) for fMRI resting-state connectivity: A multicentric validation for frontotemporal dementia**

**Sebastian Moguilner,^1,2,3^ Adolfo M. García,^1,4,5^ Ezequiel Mikulan,^1,4^ Eugenia Hesse,^1,4^ Indira García-Cordero,^1,4^ Margherita Melloni,^1,4^ Sabrina Cervetto,^1,6^ Cecilia Serrano,^7^ Eduar Herrera,^1,4,8^ Pablo Reyes,^9^ Diana Matallana,^9^ Facundo Manes,^1,4,10^ Agustín Ibáñez,^1,4,10,11,12^ *Lucas Sedeño^1,4^ .**

^1^ Laboratory of Experimental Psychology and Neuroscience (LPEN), Institute of Cognitive and Translational Neuroscience (INCYT), INECO Foundation, Favaloro University, Buenos Aires, Argentina

^2^ Fundación Escuela de Medicina Nuclear (FUESMEN) and Comisión Nacional de Energía Atómica (CNEA), Buenos Aires, Argentina

^3^ Instituto Balseiro and Facultad de Ciencias Exactas y Naturales, Universidad Nacional de Cuyo (UNCuyo), Mendoza, Argentina

^4^ National Scientific and Technical Research Council (CONICET), Av. Rivadavia 1917, C1033AAJ, Buenos Aires, Argentina

^5^ Faculty of Education, National University of Cuyo (UNCuyo), Sobremonte 74, C5500, Mendoza, Argentina

^6^ Departamento de Educación Física y Salud, Instituto Superior de Educación Física, Universidad de la República, Montevideo, Uruguay

^7^ Memory and Balance Clinic, Buenos Aires, Argentina.

^8^ Departamento de Estudios Psicológicos, Universidad ICESI, Cali, Colombia

^9^ Intellectus Memory and Cognition Center, Mental Health and Psychiatry Department, San Ignacio Hospital, Aging Institute, Pontifical Javeriana University, Bogotá, Colombia

^10^ Centre of Excellence in Cognition and its Disorders, Australian Research Council (ARC), Sydney, Australia

^11^ Center for Social and Cognitive Neuroscience (CSCN), School of Psychology, Universidad Adolfo Ibáñez, Diagonal Las Torres 2640, Santiago de Chile, Chile

^12^ Universidad Autónoma del Caribe, Calle 90, No 46-112, C2754, Barranquilla, Colombia

**Corresponding author:** Lucas Sedeño, Ph.D.

INCYT. Pacheco de Melo 1860, C1126AAB, Buenos Aires, Argentina. Phone and fax: +54 (11) 4807-4748. Mail: [lsedeno@ineco.org.ar](mailto:lsedeno@ineco.org.ar)

1. **Supplementary information 1:** Clinical details.
2. **Supplementary information 2:** Image acquisition steps.
3. **Supplementary information 3:** Image preprocessing.
4. **Supplementary information 4:** Seed analysis.
5. **Supplementary information 5:** wSDM model.
6. **Supplementary information 6:** Thresholding details.
7. **Supplementary information 7:** Statistical procedures and analysis details.
8. **Supplementary information 8:** Machine learning classifiers details.
9. **Supplementary information 9:** Consistency of resting state networks in controls.
10. **Supplementary information 10:** Partial correlations analysis.
11. **Supplementary table 1:** fMRI acquisition parameters.
12. **Supplementary table 2:** Movement parameters.
13. **Supplementary table 3:** Machine learning classifier accuracy results for Country-1 and Country-2, for R and wSDM (using the SVM classifier in the SN).
14. **Supplementary table 4:** Machine learning classifier accuracy results for Country-1 and Country-2, for R and wSDM. (using the kNN classifier in the SN).
15. **Supplementary table 5:** Seed analysis results.
16. **Supplementary table 6:** Machine learning results for Country-1 and Country-2 for R, DM and wSDM (using the SVM classifier in the DMN).
17. **Supplementary table 7:** Machine learning results for Country-1 and Country-2 for R, DM and wSDM (using the kNN classifier in the DMN).
18. **Supplementary table 8**: Machine learning results for Country-1 and Country-2 for R and wSDM (using the SVM and the kNN classifier in the Visual network).
19. **Supplementary figure 1:** wSDM flowchart.
20. **Supplementary figure 3:** DMN consistency analysis without thresholding.
21. **Supplementary figure 4:** SN consistency analysis without thresholding.
22. **Supplementary figure 8:** Visual network.
23. **Supplementary figure 9:** bvFTD vs. Controls FC in the Visual network.
24. **Supplementary figure 10**: Accuracy and ROC curves for Country-1 and Country-2, for R, DM, and wSDM (using the SVM classifier in the SN).
25. **Supplementary figure 11:** Machine learning statistical Analysis for Country-1 and Country-2 (using the SVM classifier in the SN).
26. **Supplementary figure 12:** Accuracy and ROC curves for Country-1 and Country-2, for R, DM, and wSDM (using the kNN classifier in the SN).
27. **Supplementary figure 13:** Machine learning statistical analysis for Country-1 and Country-2. (using the kNN classifier in the SN).
28. **Supplementary Information 1:** Clinical details.

Diagnosis was supported by a standard clinical examination including an extensive battery of neurological, neuropsychiatric, and neuropsychological assessments. Then, each case was individually reviewed in a multidisciplinary clinical meeting involving cognitive/behavioral neurologists, psychiatrists, and neuropsychologists. The patients were functionally impaired and exhibited prominent changes in personality and social behavior, as verified by caregivers. All patients showed frontal atrophy on MRI, and those undergoing SPECT assessment further exhibited frontal hypoperfusion. They were all in early/mild disease stages and did not fulfill criteria for specific psychiatric disorders. Patients presenting primarily with language deficits were excluded.

Each sample was matched on gender, age, and education with healthy controls from its respective center (Supplementary table 1). These participants presented no history of drug abuse, psychiatric or neurological disease.

1. **Supplementary information 2:** Image acquisition steps.

All Country-1 participants were scanned in a 1.5-T Phillips Intera scanner with a standard head coil. We used a T1-weighted spin echo sequence covering the whole brain to obtain anatomical 3D scans spin echo volumes, parallel to the plane connecting the anterior and posterior commisures. The following parameters were employed: matrix size = 256 × 240, 120 slices, voxel size = 1 x 1 x 1 mm^3^; repetition time (TR) = 7489 ms; echo time (TE) = 3420 ms; flip angle = 8°, acquisition time = 7 minutes. Country-2 participants underwent a whole-brain structural T1-rapid gradient-echo (MP RAGE) anatomical 3D scan performed in a 3T Philips Achieva scanner. Sequence parameters were: matrix size = 256 x 256, 160 slices, 1 x 1 x 1 mm^3^ isotropic; TR = 8521 ms; TE = 4130 ms; flip angle = 9º ms, acquisition time = 8 minutes.

In the resting-state protocol, all participants were asked not to think about anything in particular during scanning, to keep their eyes closed, and to avoid moving or falling sleep [^1-4^](#_ENREF_1).

1. **Supplementary information 3:** Image preprocessing.

For each preprocessing step, DPARSF called the Statistical Parametric Mapping (SPM 12**)** and the Resting-State fMRI Data Analysis Toolkit (REST V.1.7) to process the data. Before preprocessing, the first five volumes of each subject’s resting-state session were discarded to ensure steady state magnetization. Then, the images were slice-time corrected (using as reference the middle slice of each volume), and aligned to the first scan of the session to correct head movement. To reduce the effects of motion and physiological artifacts, six head-motion parameters, as well as white matter (WM) and cerebrospinal fluid signals (CFS), were removed as nuisance variables. CFS and WM masks for this procedure were derived from the tissue segmentation of each subject’s T1 scan in native space. The global signal (GS) was not considered as a nuisance variable in our preprocessing pipeline given that it is composed of both neural and non-neural signals, and several works have shown that it may artificially introduce anti-correlations in cross-regional connectivity [^5-7^](#_ENREF_5). Next, functional images were normalized to the MNI space using the echo-planar imaging (EPI) template from SPM [^8^](#_ENREF_8), and then they were smoothed with an 8-mm full-width half-maximum Gaussian kernel. Finally, data was band-pass filtered (0.01-0.08 Hz) given the relevance of slow frequency in the analysis of resting-state networks [^9^](#_ENREF_9)^,^[^10^](#_ENREF_10). As FC is affected by head motion, we excluded participants showing movements greater than 3 mm and/or rotations higher than 3º (5 bvFTD patients were thus omitted from Country-1, yielding the final sample of 20 patients) [^11^](#_ENREF_11). Then, we compared the mean translational and mean rotational parameters between groups in each country through ANOVA. No differences were found in any of the centers (see Supplementary table 3).

1. **Supplementary information 4:** Seed analysis.

First, seed analysis was used to explore the consistency of resting-state results across control samples in both countries. To this end, we assessed two well-known resting-state networks: the SN [^9^](#_ENREF_9)^,^[^12-15^](#_ENREF_12), which is typically impaired in bvFTD [^1^](#_ENREF_1)^,^[^4^](#_ENREF_4)^,^[^16-18^](#_ENREF_16), and the DMN, which is characteristically affected in AD [^19^](#_ENREF_19) but can also be compromised in bvFTD [^20^](#_ENREF_20); and the visual network, which is not typically affected in these conditions [^17^](#_ENREF_17)^,^[^21^](#_ENREF_21). The latter two networks were selected to evaluate the specificity of potential SN alterations in our target group. The latter network was selected to evaluate the specificity of potential SN alterations in our target group. We placed two bilateral seeds on cubic regions of interest (ROIs) with a size of 7x7x7 voxels [^22^](#_ENREF_22) for each network. One pair was located on the posterior cingulate cortex (PCC), a key node of the DMN [^14^](#_ENREF_14) (MNI coordinates 3,-54, 27 and -3,-54, 27); others were located on the higher visual cortex (MNI coordinates 26,-94,14 and -26,-94,14) for the visual network[^23^](#_ENREF_23), and still others were placed on the dorsal anterior cingulate cortex (dACC), a main hub of the SN [^13^](#_ENREF_13) (MNI coordinates 10, 34, 24 and -10, 34, 24). Among the different hubs that could be selected for the SN, the dACC was also chosen as a seed for this network given its association with disrupted functions in bvFTD [^24^](#_ENREF_24)^,^[^25^](#_ENREF_25), such as interoceptive-autonomic processing and stressor-associated anticipatory anxiety [^13^](#_ENREF_13). Moreover, this area has been used to characterize SN properties in healthy controls (e.g., [^26^](#_ENREF_26)), in psychiatric conditions (e.g., [^27-29^](#_ENREF_27)), in neurological diseases such as epilepsy [^30^](#_ENREF_30), and Tourette’s syndrome [^31^](#_ENREF_31), and in neurodegenerative conditions –including asymptomatic carriers of mutations in bvFTD-related genes, such as MAPT (microtubule-associated protein tau) or GRN (progranulin) mutations [^32-34^](#_ENREF_32). This evidence validates the selection of this region to perform a seed-based analysis to target the SN.

Seed maps of these networks were obtained by using R and wSDM between each seed and the remaining brain voxels for each participant. We chose seed analysis instead of Independent Component Analysis (ICA) –another common method to estimate resting-state networks [^35^](#_ENREF_35)^,^[^36^](#_ENREF_36)– because the latter is based on the decomposition of the fMRI signals into statistically independent components (rather than the estimation of association values) and precludes comparisons between FC methods.

1. **Supplementary information 5:** wSDM model.

To corroborate that the wSDM was able to capture non-linear associations, we develop a simple artificial model. One artificial time-series was modeled as a cosine function, while another one was modeled by the same cosine function but multiplied by a decreasing exponential function to consider a pure non-linear factor. This decreasing exponential constant with different levels was increased to introduce more non-linear behavior. Then, we estimated the association level between both time-series with R and wSDM, and we found that the latter outperforms R in the identification of non-linear associations (Supplementary Figure 2).


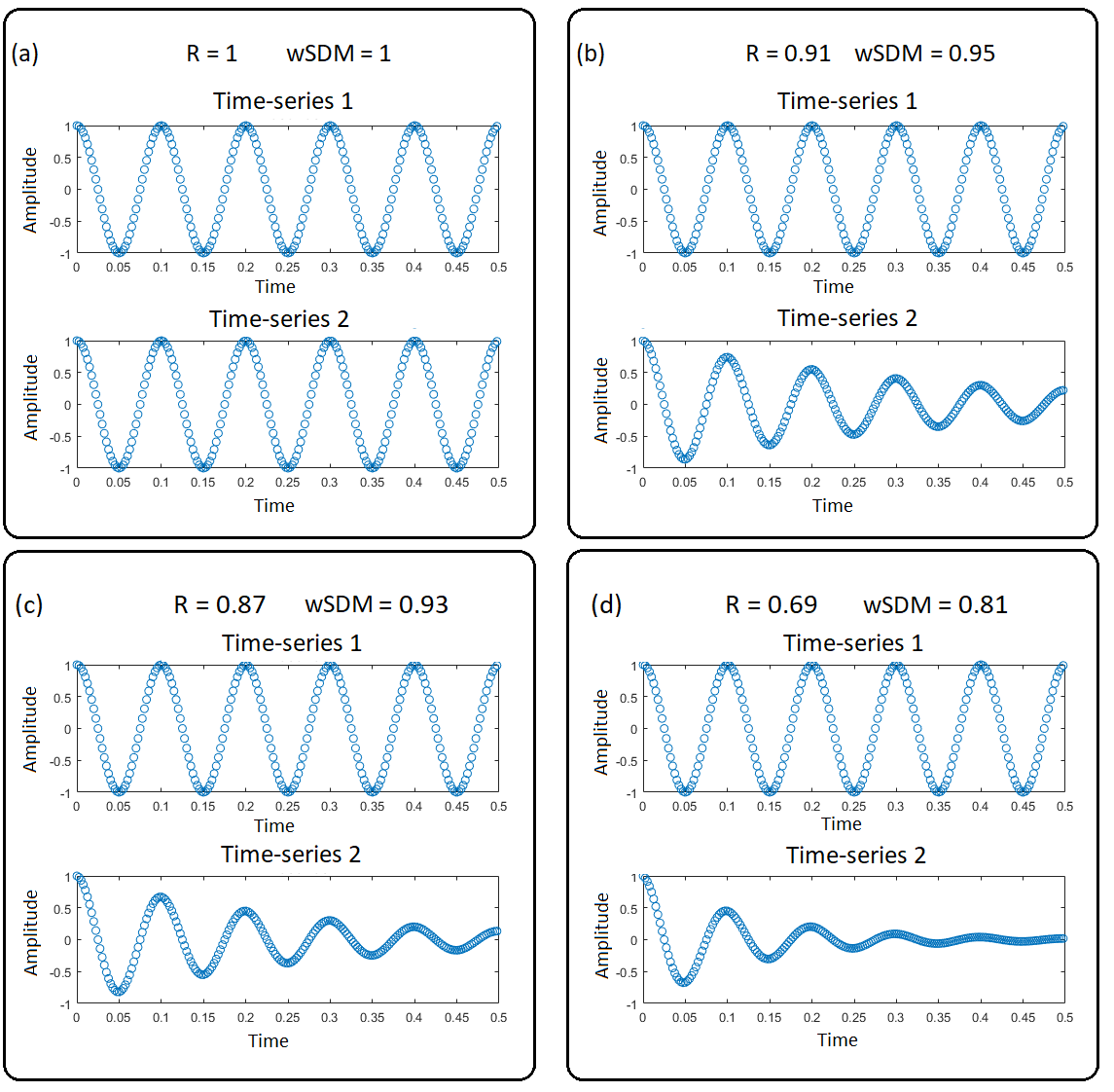


Supplementary Figure 2. Non-linear model. Panels from (a) to (d) depict four different couples of time series with an increasing non-linear factor between them.

1. **Supplementary information 6:** Thresholding details.

In order to reduce the false positive rate while studying FC, the correlation values obtained by R are usually thresholded at a fixed value [^9^](#_ENREF_9)^,^[^37^](#_ENREF_37). Yet, given that R and wSDM are expressed in different units, we employed a proportional threshold [^38^](#_ENREF_38) enabling comparisons between measures. The cut-off for this threshold was based on percentile values ranging from the 20^th^ to the 60^th^ percentile, in incremental steps of 5, keeping the values on the right tail of the distributions. This percentile range was chosen to cover a variety of the most commonly used thresholds for positive correlations [^39-41^](#_ENREF_39). This yielded a scale-free threshold that considers percentages of the most significant voxels instead of using fixed values that may be incompatible between different FC measures. Moreover, increasing the threshold actually reduces the number of features (surviving voxels), which allows evaluating machine learning classifier accuracy across different thresholds to find the optimal number of features [^42^](#_ENREF_42). The main results of this analysis are based on the 30^th^ percentile, which yielded most of the maximum classification accuracy rates on our machine learning analysis, relative to other thresholds (see section 3.3 for further details).

1. **Supplementary information 7:** Statistical procedures and analysis details.

The consistency analysis is based on a voxel-wise correlation analysis between the maps (*t*-values) of each dataset, from each comparison. As in a previous report [^4^](#_ENREF_4), we included the data from the conjunction results of each method to constrain the correlation analysis between voxels that yielded significant differences across countries. In addition, to compare this reproducibility metric between linear and non-linear measures, we tested the differences in their correlation slopes using a *t*-test [^43^](#_ENREF_43) given by the formula:

$t = \frac{b_{1}- b_{2}}{\sqrt{{{Sb}_{1}}^{2}+{{Sb}_{2}}^{2}}}$ (9)

With $b_{1}$ and $b_{2}$ as the slopes of the regression lines, while ${sb}_{1}$ and ${sb}_{2}$ as the error variances.

1. **Supplementary information 8:** Machine learning classifiers details.

To identify the most robust association method to discriminate bvFTD patients from controls, we employed machine learning classifiers for the data of each center. There are mainly two approaches to estimate class boundaries: one involves creating a linear hyperplane (i.e., linear decision boundary) and the other a more complex shape with different distance metrics (i.e., non-linear decision boundary) [^42^](#_ENREF_42). To avoid a-priori assumptions regarding the shape of the class boundaries, we chose to assess the classification performance using both approaches. First, we applied SVMs with a linear kernel, one of the most widely used methods for neuroimaging data classification given that it can effectively handle high-dimensional data while providing good classification results [^44^](#_ENREF_44). The SVM classifier has been successfully employed in fMRI connectivity studies using seed analysis [^45^](#_ENREF_45)^,^[^46^](#_ENREF_46). For classification purposes, a hyperplane in the feature space is determined by maximizing the margin of separation between the two classes given finite set of learning patterns. Once that the optimum hyperplane is found, by sweeping the feature space in a direction orthogonal to the hyperplane, we can calculate, for different points, the classification’s true positive rate (TPR) and false positive rate (FPR). Then, we can plot a receiver output characteristic (ROC) curve to measure the performance of the classifier by calculating the area under the curve (AUC). Finally, to test for nonlinear boundaries, we employed the k-nearest neighbor (kNN) classifier (a non-linear boundary classifier) to classify objects based on their proximity to the training samples relative to k-nearest neighbors. Choosing the optimum k-number is a major issue, depending on multiple factors [^47^](#_ENREF_47). Hence, to avoid overfitting when comparing different FC measures, we choose k = 1 for all measures to evaluate classification accuracy. This nearest neighbor classifier has been also successfully employed in the classification of fMRI patterns [^48^](#_ENREF_48). To construct the ROC curve in this case, we varied a threshold based on the majority class label [^42^](#_ENREF_42).

We employed the SVM and KNN classifiers included in the CoSMoMVPA [^49^](#_ENREF_49) toolbox using default settings; we applied a linear kernel and the box constraint soft-margin C parameters for SVM, where misclassifications have the cost of a penalty factor C, and the Euclidean distance for kNN. To face the problem that fMRI features widely exceed the training examples in classification analysis (i.e., almost a million voxels), it is recommended to reduce the number of features using a particular ROI [^42^](#_ENREF_42). To this end, we used a standard SN mask, reported in [^50^](#_ENREF_50), which encompassed the anterior insula and the dorsal ACC. To test for the specificity of this network, we also compared the classification accuracy of the DMN [(using a standard mask, reported in [^50^](#_ENREF_50), spanning the PCC and the medial prefrontal cortex (mPFC)]. For a robust estimation of classification performance, we employed the leave-one-out cross-validation (LOOCV) technique where one subject (i.e., a patient or a control) is used for prediction, and the remaining subjects are employed as the training data. This process is repeated as many times as needed such that each subject in the sample is used once as the validation data [^44^](#_ENREF_44). To assess which measure better classified bvFTD and healthy controls, we compared the classification accuracy rates and the AUC between R and wSDM using both classifiers. To test for significant differences between the classifications rates of the measures under all thresholds, we employed the Wilcoxon test. This non-parametric statistical test was implemented by comparing all the obtained classification accuracies for each measure and for each classifier. To test for the statistical significance of the results under each measure’s optimal threshold, a null distribution of AUC values was obtained by shuffling the classes label’s (i.e., Healthy Control and bvFTD). Thus, an empirical *p-*value could be calculated [^51^](#_ENREF_51).

1. **Supplementary information 9:** Consistency of resting state networks in controls.

To compare the extent of the DMN and SN seed-based maps at the individual level, we first set to 1 all the connectivity values above the 30th percentile (considering the values from the right tail of the distribution), assigning a 0 to all other values. Then, we summed each of these individual voxel-wise binarized maps to obtain an overlap result that illustrates the homogeneous extension of the resting-state network in each sample –i.e., the higher the value of the overlap map in a voxel, the greater the consistency of the network across individuals. In the control groups, we found –based on a two sample t-test with two tails–, that the wSDM presented significant higher values on the overlap maps for each network compared to R [for the DMN, Country-1: t-value = 7.62, p < .001, and Country-2: t-value = 4.72, p < .001; for the SN, Country-1: t-value = 4.97, p < .001, and Country-2: t-value = 5.21, p < .001].The same was true for the bvFTD patients, but only as regards the DMN [Country-1: t-value = 6.45, p < .001, and Country-2: t-value = 11.77, p < .001] (see Supplementary figure 5). In the SN, the last group presented overlap maps with very low values [no significant difference was found between methods, for Country-1: t-value = 1.58, p = .011, and for Country-2: t-value = 2.95, p = .009]; this heterogeneous result is consistent with the alteration of this network in bvFTD [^17^](#_ENREF_17)^,^[^21^](#_ENREF_21) (see Supplementary figure 6). This finding underscores the greater robustness of wSDM over R across individuals. Briefly, this evidence corroborates that our non-linear measure outperforms R as a reliable and consistent method to identify and characterize functional connectivity networks.


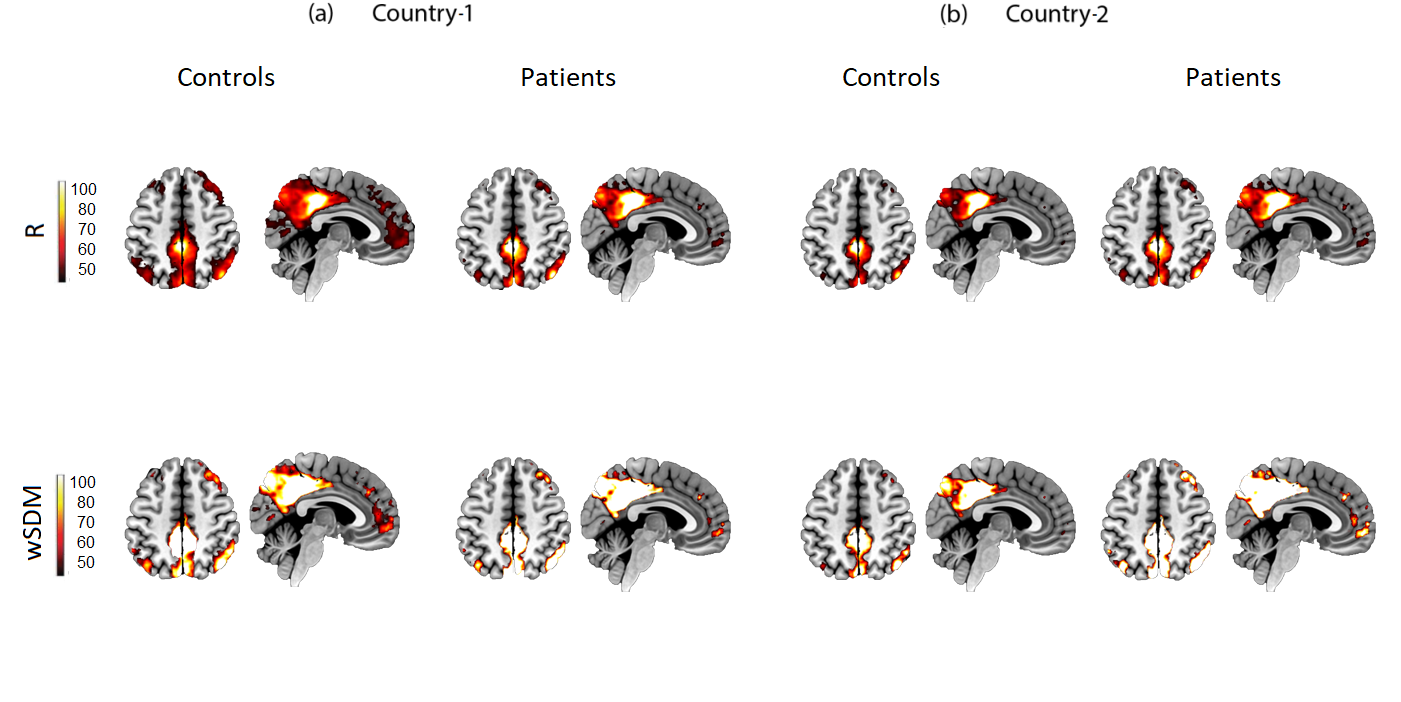


Supplementary figure 5. DMN extension at the individual level. To achieve comparability across countries –given that each center has different number of participants−, overlap maps show the voxels in which we found more than 50% of consistent connections above the 30^th^ percentile (considering the values from the right tail of the distribution) across participants. Axial plane z = 48, sagittal plane x = 4. All brain images are presented according to neurological convention.


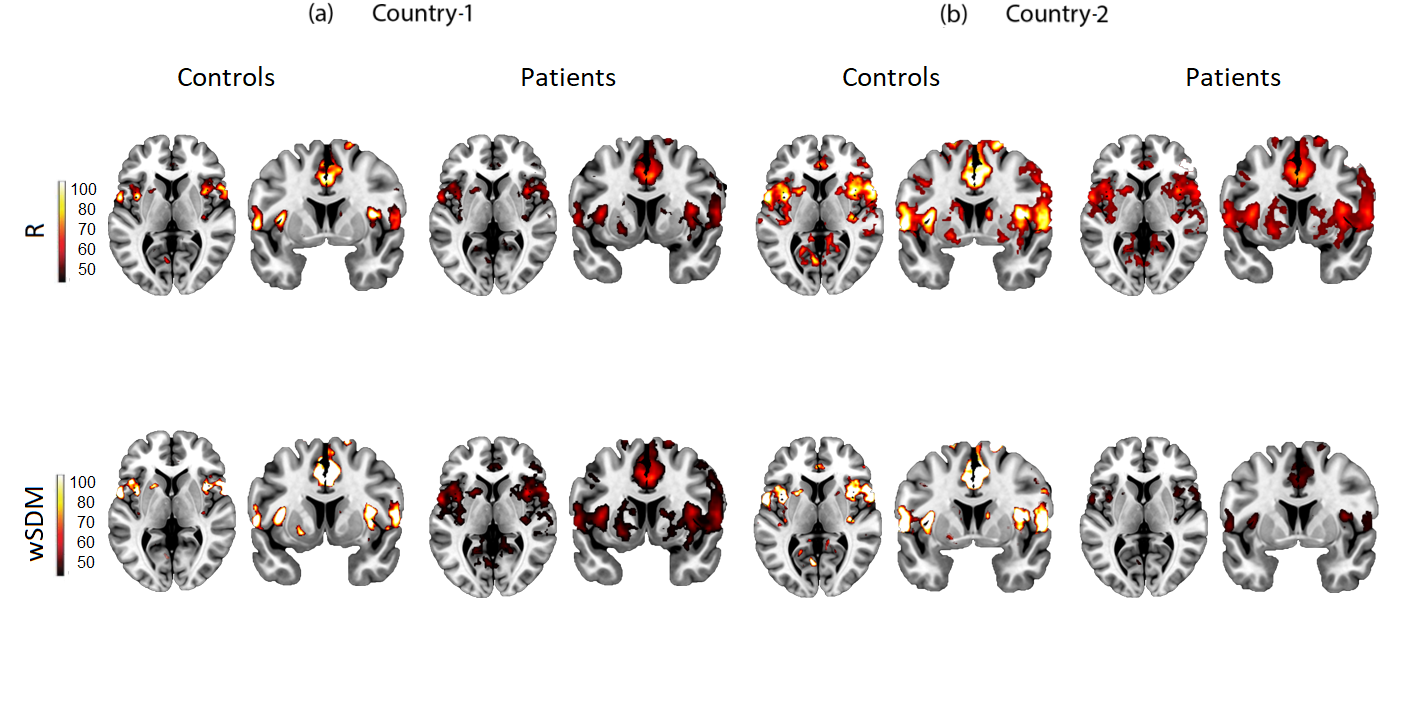
 Supplementary figure 6. SN extension at the individual level. To achieve comparability across countries –given that each center has different number of participants−, overlap maps show the voxels in which we found more than 50% of consistent connections above the 30^th^ percentile (considering the values from the right tail of the distribution) across participants. Axial plane z = 3, sagittal plane y = 6. All brain images are presented according to neurological convention.

1. **Supplementary information 10:** Partial correlations analysis.

To select the SN node to compare partial correlations (PC) against wSDM, we used the bilateral anterior cingulate cortex (ACC) and the insular region from the Automated Anatomical Labeling (AAL) atlas (Tzourio-Mazoyer, Landeau et al. 2002). Partial correlations and wSDM within the regions of the SN were tested with the same classification methods applied for our seed-based analysis. We found low classification accuracy results when compared to the voxel-based analysis (Supplementary Table 8). Based on a pairwise statistical comparison of ROC curves, we found that wSDM significantly surpassed both R and PC results in its classification rates for both countries [For Country-1 and the SVM classifier: R vs. PC z-statistic = 2.34, p = .01; PC vs. wSDM z-statistic = 1.45, p = .03; R vs. wSDM z-statistic = 2.46, p = .01. For Country-2 and the SVM classifier: R vs. PC z-statistic = 3.37, p = .01, PC vs. wSDM z-statistic = 2.45, p = .02; R vs. wSDM z-statistic = 3.65, p = .009. For Country-1 and the KNN classifier: R vs. PC z-statistic = 2.94, p = .01; PC vs. wSDM z-statistic = 2.35, p = .01; R vs. wSDM z-statistic = 2.97, p = .01. For Country-2 and the KNN classifier: R vs. PC z-statistic = 1.95, p = .03, PC vs. wSDM z-statistic = 2.14, p = .03, R vs. wSDM: z-statistic =2.63, p = .02] (Supplementary Figure 7).

Supplementary Table 9. SN classification based on areas from the AAL atlas.

|  | **Country-1** | | | **Country-2** | | |
| --- | --- | --- | --- | --- | --- | --- |
| **Classifier** | **R**  **[%]** | **PC**  **[%]** | **wSDM**  **[%]** | **R**  **[%]** | **PC**  **[%]** | **wSDM**  **[%]** |
| SVM | 62.5 | 65 | 67.5 | 59.1 | 63.6 | 65.9 |
| KNN | 62.5 | 62.5 | 67.5 | 61.4 | 63.6 | 65.9 |

[%]: Accuracy rate.

SVM: Linear kernel Support Vector Machines classifier.

KNN: K-Nearest Neighbors classifier.

R: Pearson Correlation.

PC: Partial Correlation.

wSDM: Weighted Symbolic Dependence Metric


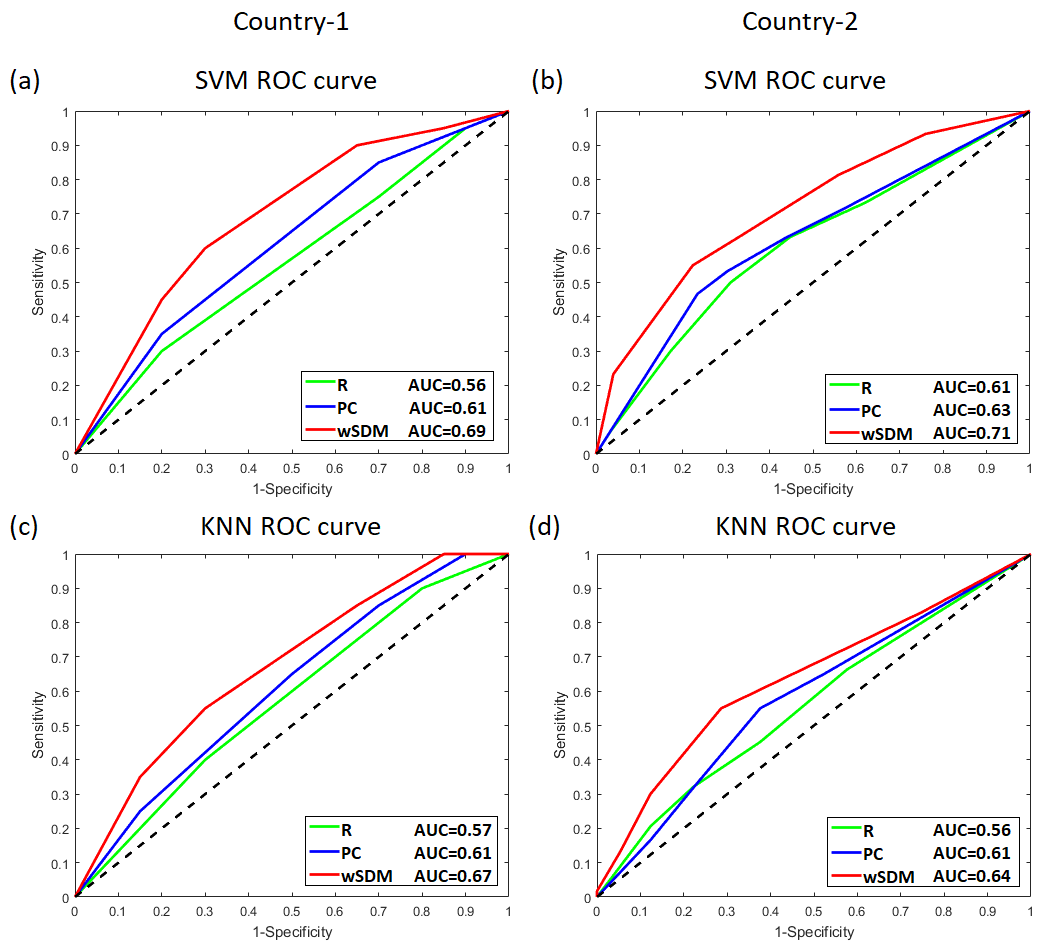


Supplementary Figure 7. (a)-(b) SVM ROC curves graph for Country-1 and Country-2. (c)-(d) KNN ROC curves for Country-1 and Country-2. SVM: Linear kernel Support Vector Machines classifier. KNN: K-Nearest Neighbors classifier. R: Pearson Correlation. PC: Partial Correlation. wSDM: Weighted Symbolic Dependence Metric.

1. **Supplementary table 1:** fMRI acquisition parameters.

|  | **Country-1** | **Country-2** |
| --- | --- | --- |
| Firm | Philips Intera | Philips Achieva |
| Tesla | 1.5 T | 3 T |
| Nº slices | 33 slices | 40 slices |
| Voxel size | 3.6 mm x 3.6 mm x 4 mm | 3 mm x 3 mm x 3 mm |
| Flip angle | 90º | 90º |
| Acquisition | Ascending. Parallel to the anterior and posterior commissures. | Ascending. Parallel to the anterior and posterior commissures. |
| Repetition time (TR) | 2777 ms | 3000 ms |
| Echo time (TE) | 50 ms | 30 ms |
| Duration | 10 min | 5 min |
| Instruction | “Do not think about anything in particular”. | “Do not think about anything in particular”. |
| Nº volumes | 209 | 120 |

1. **Supplementary table 2:** Movement parameters.

| **Movement parameters** | | | | | | | | |
| --- | --- | --- | --- | --- | --- | --- | --- | --- |
|  | **Country-1** | | | | **Country-2** | | | |
|  | **C** | **bvFTD** | ***p-*values** | ***F-*values** | **C** | **bvFTD** | ***p-*values** | ***F-*values** |
| Mean translational (mm) | 0.07 (0.05) | 0.09 (0.07) | .22 | 0.56 | 0.05 (0.03) | 0.05 (0.02) | .31 | 0.64 |
| Mean rotational (º) | 0.1 (0.08) | 0.7 (0.03) | .18 | 0.42 | 0.03 (0.01) | 0.04 (0.02) | .20 | 0.55 |

Mean (SD)

1. **Supplementary table 3:** Machine learning classifier accuracy results for Country-1 and Country-2, for R and wSDM (using the SVM classifier in the SN).

|  | **Country-1** | | | **Country-2** | | |
| --- | --- | --- | --- | --- | --- | --- |
| **Threshold**  **[Percentile]** | **R**  **[%]** | **DM**  **[%]** | **wSDM**  **[%]** | **R**  **[%]** | **DM**  **[%]** | **wSDM**  **[%]** |
| 20^th^ | 60 | 65 | 67.5 | 79.5 | 84.1 | 86.4 |
| 25^th^ | 62.5 | 67.5 | 70 | 81.8 | 86.4 | 88.6 |
| 30^th^ | 62.5 | 65 | 70 | 84.1 | 88.6 | 88.6 |
| 35^th^ | 62.5 | 65 | 67.5 | 81.8 | 84.1 | 86.4 |
| 40^th^ | 57.5 | 65 | 67.5 | 79.5 | 81.8 | 84.1 |
| 45^th^ | 57.5 | 65 | 65 | 79.5 | 81.8 | 84.1 |
| 50^th^ | 57.5 | 62.5 | 65 | 72.7 | 79.5 | 79.5 |
| 55^th^ | 47.5 | 62.5 | 62.5 | 61.4 | 77.3 | 77.3 |
| 60^th^ | 47.5 | 57.5 | 57.5 | 61.4 | 77.3 | 77.3 |

[%]: Accuracy rate.

SVM: Support Vector Machines classifier.

R: Pearson Correlation Coefficient.

DM: Dependence Measure (unweighted symbolic ${I\phi}^{2}$).

WSDM: Symbolic Weighted Dependence Metric.

1. **Supplementary table 4:** Machine learning classifier accuracy results for Country-1 and Country-2, for R and wSDM. (using the kNN classifier in the SN).

|  | **Country-1** | | | **Country-2** | | |
| --- | --- | --- | --- | --- | --- | --- |
| **Threshold**  **[Percentile]** | **R**  **[%]** | **DM**  **[%]** | **wSDM**  **[%]** | **R**  **[%]** | **DM**  **[%]** | **wSDM**  **[%]** |
| 20^th^ | 72.5 | 77.5 | 77.5 | 59.1 | 56.8 | 63.6 |
| 25^th^ | 75 | 77.5 | 80 | 59.1 | 59.1 | 70.5 |
| 30^th^ | 75 | 80 | 80 | 61.4 | 61.4 | 70.5 |
| 35^th^ | 75 | 77.5 | 80 | 61.4 | 61.4 | 65.9 |
| 40^th^ | 75 | 77.5 | 77.5 | 61.4 | 61.4 | 65.9 |
| 45^th^ | 72.5 | 75 | 77.5 | 61.4 | 59.1 | 63.6 |
| 50^th^ | 72.5 | 75 | 75 | 61.4 | 56.8 | 61.4 |
| 55^th^ | 72.5 | 72.5 | 75 | 59.1 | 54.5 | 61.4 |
| 60^th^ | 72.5 | 72.5 | 72.5 | 59.1 | 56.8 | 61.4 |

[%]: Accuracy rate.

SVM: Support Vector Machines classifier.

R: Pearson Correlation Coefficient.

DM: Dependence Measure (unweighted symbolic ${I\phi}^{2}$).

wSDM: Symbolic Weighted Dependence Metric.

1. **Supplementary table 5:** Seed analysis results.

| **COUNTRY-1 - CONTROLS** | | | | | | | |
| --- | --- | --- | --- | --- | --- | --- | --- |
| **R DMN** | | | | | | | |
| **Cluster nº** | **Cluster *p* (FWE)** | **Peak *p* (FWE)** | **Peak *T*** | **X (mm)** | **Y (mm)** | **Z (mm)** | **Area** |
| 1 | > .001 | > .001 | 22 | -5 | -51 | 34 | Left posterior cingulate cortex |
| 2 | > .001 | > .001 | 6.91 | 40 | -73 | 48 | Right angular gyrus |
| **wSDM DMN** | | | | | | | |
| 1 | > .001 | > .001 | 45.13 | 0 | -37 | 46 | Right dorsal posterior cingulate cortex |
| 2 | > .001 | > .001 | 20.79 | 40 | -73 | 48 | Right angular gyrus |
|  |  | > .001 | 15.61 | 34 | -81 | 30 | Right angular gyrus |
| 3 | > .001 | > .001 | 12.77 | -48 | -43 | 30 | Left angular gyrus |
|  |  | > .001 | 10.77 | -50 | -35 | 30 | Left supramarginal gyrus |
|  |  | > .001 | 9.97 | -55 | -43 | 32 | Left angular gyrus |
| 4 | > .001 | > .001 | 8.20 | -5 | 61 | 0 | medial Prefrontal Cortex |
| **R SN** | | | | | | | |
| 1 | > .001 | > .001 | 13.9 | 0 | 9 | 40 | Dorsal anterior cingulate |
| **wSDM SN** | | | | | | | |
| 1 | > .001 | > .001 | 42.80 | -58.5 | -10.5 | -10.5 | Dorsal anterior cingulate |
| 2 | > .001 | > .001 | 26.52 | 36 | 20 | 1 | Right Insula |
| 3 | > .001 | > .001 | 11.31 | -42 | 12 | 6 | Left inferior frontal operculum |
| **R VN** | | | | | | | |
| 1 | > .001 | > .001 | 19.77 | 26 | -92 | 14 | Right visual association cortex |
| 2 | > .001 | > .001 | 26.54 | -25 | -93 | 15 | Left visual association cortex |
| **wSDM VN** | | | | | | | |
| 1 | > .001 | > .001 | 24.21 | 28 | -96 | 15 | Right visual association cortex |
| 2 | > .001 | > .001 | 29.36 | -23 | -95 | 12 | Left visual association cortex |
| **COUNTRY-2 - CONTROLS** | | | | | | | |
| **R DMN** | | | | | | | |
| **Cluster nº** | **Cluster *p* (FWE)** | **Peak *p* (FWE)** | **Peak *T*** | **X (mm)** | **Y (mm)** | **Z (mm)** | **Area** |
| 1 | > .001 | > .001 | 35.17 | 2 | -43 | 32 | Posterior cingulate cortex |
| 2 | > .001 | > .001 | 22.42 | 44 | -71 | 46 | Right angular gyrus |
|  | > .001 | > .001 | 18.41 | 44 | -59 | 38 | Right angular gyrus |
| 3 | > .001 | > .001 | 17.63 | -38 | -83 | 40 | Left angular gyrus |
|  | > .001 | > .001 | 15.98 | -46 | -83 | 28 | Left angular gyrus |
| **wSDM DMN** | | | | | | | |
| 1 | > .001 | > .001 | 35.43 | 2 | -43 | 32 | Posterior cingulate cortex |
| 2 | > .001 | > .001 | 22.37 | 44 | -69 | 48 | Right angular gyrus |
|  |  | > .001 | 19.51 | 44 | -57 | 38 | Right angular gyrus |
|  |  | > .001 | 17.50 | 58 | -59 | 30 | Right angular gyrus |
| 3 | > .001 | > .001 | 15.19 | -54 | -53 | 48 | Left angular gyrus |
|  |  | > .001 | 14.86 | -42 | -75 | 42 | Left angular gyrus |
|  |  | > .001 | 13.95 | -44 | -83 | 28 | Left angular gyrus |
| 4 | > .001 | > .001 | 14.60 | 4 | 49 | 10 | medial prefrontal cortex |
| **R SN** | | | | | | | |
| 1 | > .001 | > .001 | 11.41 | 5 | 21 | 42 | Dorsal anterior cingulate |
|  | > .001 | > .001 | 7.31 | 4 | 15 | 34 | Dorsal anterior cingulate |
| **wSDM SN** | | | | | | | |
| 1 | > .001 | > .001 | 22.13 | 5 | 21 | 42 | Dorsal anterior cingulate |
| 2 | > .001 | > .001 | 20.27 | 40 | 3 | 12 | Right insula |
| 3 | > .001 | > .001 | 16.85 | -36 | -5 | 3 | Left insula |
| 4 | > .001 | > .001 | 16.11 | -58 | 3 | 4 | Left inferior frontal operculum |
| **R VN** | | | | | | | |
| 1 | > .001 | > .001 | 16.59 | 26 | -94 | 14 | Right visual association cortex |
| 2 | > .001 | > .001 | 24.92 | -26 | -96 | 13 | Left visual association cortex |
| **wSDM VN** | | | | | | | |
| 1 | > .001 | > .001 | 22.36 | 27 | -94 | 15 | Right visual association cortex |
| 2 | > .001 | > .001 | 29.96 | -25 | -95 | 13 | Left visual association cortex |
| **CONJUNCTION ANALYSIS - CONTROLS** | | | | | | | |
| **R DMN** | | | | | | | |
| **Cluster nº** | **Cluster *p* (FWE)** | **Peak *p* (FWE)** | **Peak *T*** | **X (mm)** | **Y (mm)** | **Z (mm)** | **Area** |
| 1 | > .001 | > .001 | 15.25 | 0 | -27 | 46 | Posterior cingulate cortex |
| 2 | > .001 | > .001 | 12.14 | 44 | -71 | 46 | Right angular gyrus |
|  | > .001 | > .001 | 10.15 | 44 | -59 | 38 | Right angular gyrus |
| **wSDM DMN** | | | | | | | |
| 1 | > .001 | > .001 | 18.15 | 4 | -53 | 30 | Posterior cingulate cortex |
| 2 | > .001 | > .001 | 17.02 | 42 | -71 | 48 | Right angular gyrus |
|  |  | > .001 | 13.41 | 46 | -73 | 38 | Right angular gyrus |
|  |  | > .001 | 12.58 | 50 | -57 | 52 | Right angular gyrus |
| 3 | > .001 | > .001 | 11.41 | -40 | -75 | 46 | Left angular gyrus |
|  |  | > .001 | 10.81 | -38 | -59 | 54 | Left angular gyrus |
|  |  | > .001 | 10.35 | -45 | -79 | 54 | Left angular gyrus |
| 4 | > .001 | > .001 | 5.60 | 4 | 49 | 10 | medial prefrontal cortex |
| **R SN** | | | | | | | |
| 1 | > .001 | > .001 | 5.62 | 5 | 21 | 42 | Dorsal anterior cingulate |
| **wSDM SN** | | | | | | | |
| 1 | > .001 | > .001 | 16.53 | -4 | 7 | 44 | Dorsal anterior cingulate |
| 2 | > .001 | > .001 | 9.38 | 38 | 13 | 4 | Right inferior frontal operculum |
| 3 | > .001 | > .001 | 9.49 | -48 | 15 | 4 | Left operculum |
| **R VN** | | | | | | | |
| 1 | > .001 | > .001 | 17.23 | 27 | -92 | 14 | Right visual association cortex |
| 2 | > .001 | > .001 | 23.12 | -25 | -95 | 13 | Left visual association cortex |
| **wSDM VN** | | | | | | | |
| 1 | > .001 | > .001 | 18.42 | 25 | -95 | 15 | Right visual association cortex |
| 2 | > .001 | > .001 | 26.84 | -26 | -93 | 14 | Left visual association cortex |
| **COUNTRY-1 (HC>bvFTD)** | | | | | | | |
| **R SN** | | | | | | | |
| 1 | > .001 | > .001 | 9.38 | 0 | 25 | 40 | Anterior insular cortex |
| 2 | > .001 | > .001 | 4.01 | 31 | 20 | 2 | Right anterior insula |
| 3 | > .001 | > .001 | 3.25 | -42 | 12 | 6 | Left fronto-temporal operculum |
| 4 | > .001 | > .001 | 2.07 | -36 | -6 | 2 | Left insula |
| **wSDM SN** | | | | | | | |
| 1 | > .001 | > .001 | 6.73 | 2 | 21 | 15 | Anterior cingulate cortex |
| 2 | > .001 | > .001 | 6.14 | -36 | -5 | 3 | Left insula |
| 3 | > .001 | > .001 | 5.37 | 36 | 20 | 1 | Right insula |
| 4 | > .001 | > .001 | 5.51 | 58 | 3 | 4 | Right inferior frontal operculum |
| 5 | > .001 | > .001 | 5.45 | -58 | 3 | 4 | Left inferior frontal operculum |
| **R DMN** | | | | | | | |
| 1 | > .001 | > .001 | 6.87 | 1 | -27 | 46 | Posterior cingulate cortex |
| **wSDM DMN** | | | | | | | |
| 1 | > .001 | > .001 | 7.54 | 0 | -27 | 45 | Posterior cingulate cortex |
| 2 | > .001 | > .001 | 5.61 | 43 | -72 | 45 | Right angular gyrus |
| **R VN** | | | | | | | |
| 1 | > .001 | > .001 | 3.23 | 27 | -94 | 14 | Right visual association cortex |
| 2 | > .001 | > .001 | 4.74 | -26 | -95 | 13 | Left visual association cortex |
| **wSDM VN** | | | | | | | |
| 1 | > .001 | > .001 | 3.95 | 27 | -94 | 14 | Right visual association cortex |
| 2 | > .001 | > .001 | 4.66 | -26 | -95 | 13 | Left visual association cortex |
| **COUNTRY-2 (HC > bvFTD)** | | | | | | | |
| **R SN** | | | | | | | |
| 1 | > .001 | > .001 | 5.21 | 0 | 28 | 40 | Anterior cingulate cortex |
| 2 | > .001 | > .001 | 4.97 | -56 | 15 | -2 | Left insula |
| 3 | > .001 | > .001 | 5.47 | -56 | -1 | 0 | Left fronto-temporal operculum |
| **wSDM SN** | | | | | | | |
| 1 | > .001 | > .001 | 5.45 | 0 | 29 | 40 | Anterior cingulate cortex |
| 2 | > .001 | > .001 | 5.93 | -56 | 15 | 1 | Left insula |
| 3 | > .001 | > .001 | 5.73 | -58 | 3 | 2 | Left operculum |
| 4 | > .001 | > .001 | 5.04 | 38 | 11 | 4 | Right insula |
| **R DMN** | | | | | | | |
| 1 | > .001 | > .001 | 6.21 | 1 | -27 | 46 | Posterior cingulate cortex |
| 3 | > .001 | > .001 | 5.23 | -44 | -59 | 38 | Leftt angular gyrus |
| **wSDM DMN** | | | | | | | |
| 1 | > .001 | > .001 | 6.54 | 4 | 49 | 10 | medial prefrontal cortex |
| **R VN** | | | | | | | |
| 1 | > .001 | > .001 | 3.21 | 25 | -93 | 15 | Right visual association cortex |
| **wSDM VN** | | | | | | | |
| 1 | > .001 | > .001 | 4.25 | 26 | -94 | 14 | Right visual association cortex |

1. **Supplementary table 6:** Machine learning results for Country-1 and Country-2 for R, DM and wSDM (using the SVM classifier in the DMN).

|  | **Country-1** | | | **Country-2** | | |
| --- | --- | --- | --- | --- | --- | --- |
| **Threshold**  **[Percentile]** | **R**  **[%]** | **DM**  **[%]** | **wSDM**  **[%]** | **R**  **[%]** | **DM**  **[%]** | **wSDM**  **[%]** |
| 20^th^ | 45 | 55 | 67.5 | 68.2 | 68.2 | 68.2 |
| 25^th^ | 47.5 | 60 | 67.5 | 68.2 | 70.5 | 70.5 |
| 30^th^ | 50 | 57.5 | 67.5 | 65.9 | 70.5 | 72.7 |
| 35^th^ | 47.5 | 55 | 65 | 68.2 | 68.2 | 70.5 |
| 40^th^ | 47.5 | 52.5 | 60 | 65.9 | 68.2 | 68.2 |
| 45^th^ | 52.5 | 45 | 52.5 | 68.2 | 65.9 | 68.2 |
| 50^th^ | 55 | 47.5 | 47.5 | 56.8 | 68.2 | 68.2 |
| 55^th^ | 55 | 52.5 | 47.5 | 56.8 | 68.2 | 70.5 |
| 60^th^ | 55 | 52.5 | 52.5 | 56.8 | 68.2 | 68.2 |

[%]: Accuracy rate.

SVM: Support Vector Machines classifier.

R: Pearson Correlation Coefficient.

DM: Dependence Measure (unweighted symbolic ${I\phi}^{2}$).

wSDM: Symbolic Weighted Dependence Metric.

1. **Supplementary table 7:** Machine learning results for Country-1 and Country-2 for R, DM and wSDM (using the kNN classifier in the DMN).

|  | **Country-1** | | | **Country-2** | | |
| --- | --- | --- | --- | --- | --- | --- |
| **Threshold**  **[Percentile]** | **R**  **[%]** | **DM**  **[%]** | **wSDM**  **[%]** | **R**  **[%]** | **DM**  **[%]** | **wSDM**  **[%]** |
| 20^th^ | 57.5 | 57.5 | 52.5 | 45.5 | 47.7 | 50 |
| 25^th^ | 52.5 | 57.5 | 52.5 | 45.5 | 47.7 | 50 |
| 30^th^ | 52.5 | 55 | 55 | 45.5 | 45.5 | 43.2 |
| 35^th^ | 50 | 55 | 52.5 | 59.1 | 45.5 | 43.2 |
| 40^th^ | 50 | 55 | 52.5 | 52.3 | 47.7 | 45.5 |
| 45^th^ | 47.5 | 52.5 | 55 | 45.5 | 40.9 | 40.9 |
| 50^th^ | 52.5 | 50 | 50 | 53.2 | 45.5 | 43.2 |
| 55^th^ | 47.5 | 50 | 50 | 45.5 | 45.5 | 43.2 |
| 60^th^ | 52.5 | 50 | 50 | 45.5 | 40.9 | 43.2 |

[%]: Accuracy rate.

SVM: Support Vector Machines classifier.

R: Pearson Correlation Coefficient.

DM: Dependence Measure (unweighted symbolic ${I\phi}^{2}$).

wSDM: Symbolic Weighted Dependence Metric.

1. **Supplementary table 8:** Machine learning results for Country-1 and Country-2 for R and wSDM (using the SVM and the kNN classifier in the Visual network).

|  | **Linear SVM** | | | | **KNN** | | | |
| --- | --- | --- | --- | --- | --- | --- | --- | --- |
|  | **Country-1** | | **Country-2** | | **Country-1** | | **Country-2** | |
| **Threshold**  **[Percentile]** | **R**  **[%]** | **wSDM**  **[%]** | **R**  **[%]** | **wSDM**  **[%]** | **R**  **[%]** | **wSDM**  **[%]** | **R**  **[%]** | **wSDM**  **[%]** |
| 20th | 45 | 67.5 | 50 | 56.8 | 45 | 54.8 | 54.5 | 54.8 |
| 25th | 47.5 | 67.5 | 50 | 54.5 | 47.5 | 56.8 | 56.8 | 56.8 |
| 30th | 50 | 67.5 | 52.2 | 54.5 | 50 | 56.8 | 54.5 | 56.8 |
| 35th | 47.5 | 52.5 | 50 | 56.8 | 67.5 | 54.5 | 56.8 | 54.5 |
| 40th | 47.5 | 60 | 52.2 | 56.8 | 47.5 | 59.1 | 56.8 | 59.1 |
| 45th | 52.5 | 52.5 | 56.8 | 56.8 | 52.5 | 59.1 | 52.2 | 59.1 |
| 50th | 55 | 47.5 | 56.8 | 59.1 | 55 | 59.1 | 56.8 | 59.1 |
| 55th | 55 | 52.5 | 56.8 | 59.1 | 47.5 | 56.8 | 50 | 56.8 |
| 60th | 55 | 52.5 | 59 | 56.8 | 55 | 47.5 | 54.5 | 56.8 |

[%]: Accuracy rate.

Linear SVM: Linear kernel Support Vector Machines classifier.

1. **Supplementary figure 1:** wSDM flowchart.


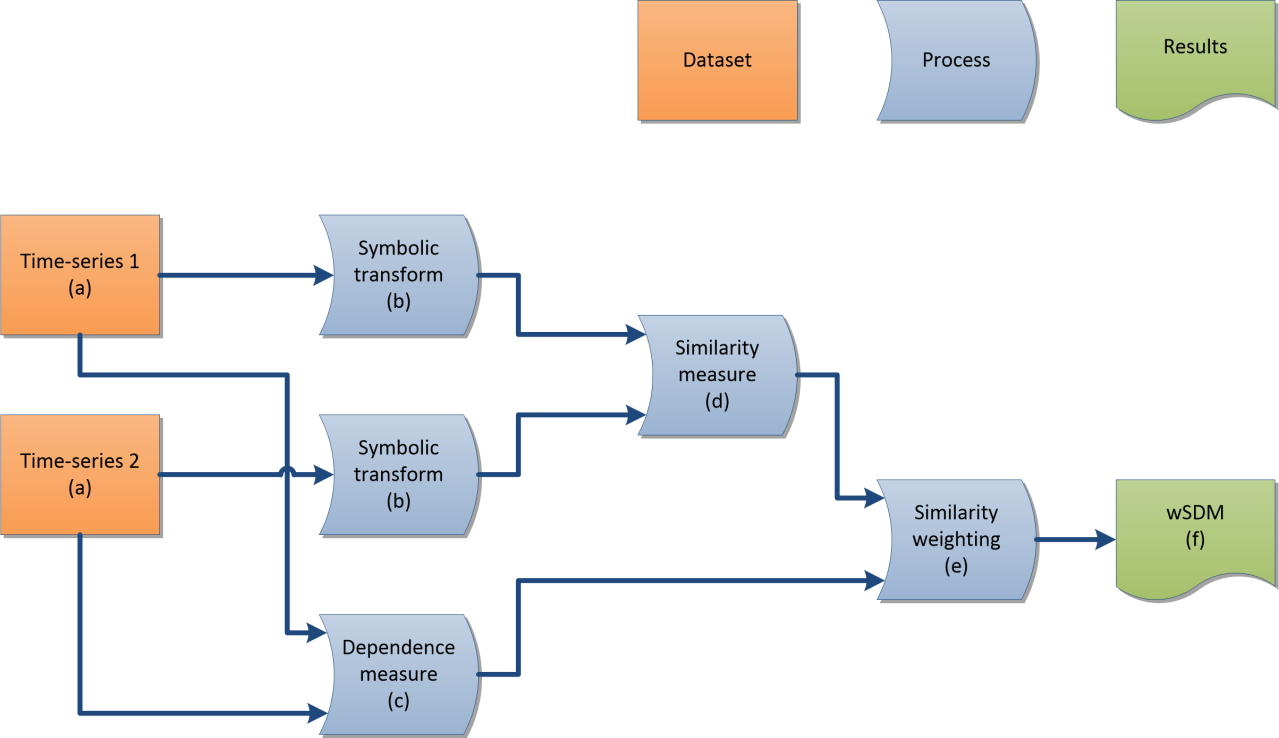


Supplementary Figure 1. Flowchart showing the sequence of calculations involved in the wSDM. (a) Two time-series are obtained from the voxels or areas for the analysis. (b) The Hoeffding Phi Square is used to analyze the dependency of the time-series. (c) The time-series are transformed into symbols. (d) The Hamming similarity measure is applied to estimate the similarity between the transformed time-series. (e) Finally, the result of the dependence measure is weighted to produce the wSDM (f).

1. **Supplementary figure 3:** DMN consistency analysis without thresholding.


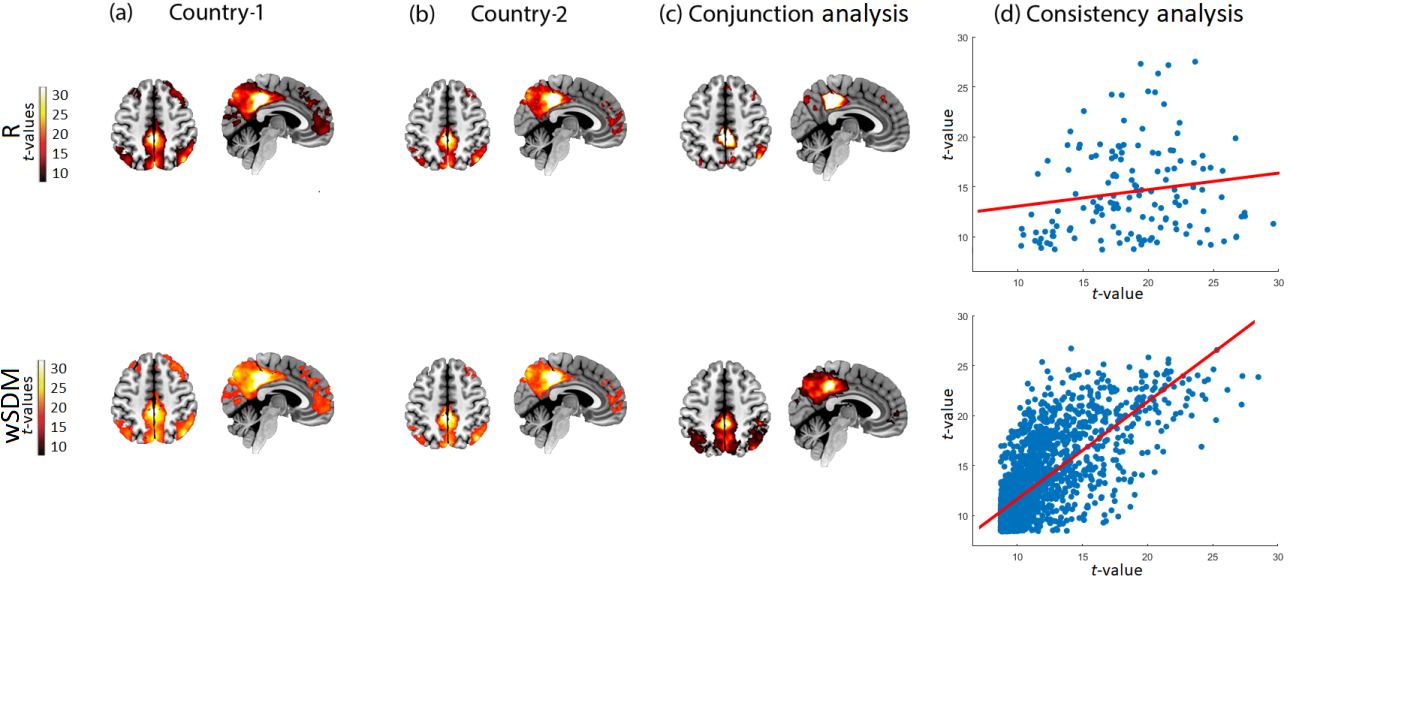


Supplementary Figure 3. DMN consistency analysis without thresholding. (a)-(b). Seed analysis results of the DMN using R and wSDM (FWE-corrected, *p* = .05 on the voxel level, extent threshold = 30) (axial plane z = 48, sagittal plane x = 4). (c) Cluster overlap between Country-1 and Country-2 (FWE-corrected, *p* = .05, extent threshold = 30 voxels). (d) Consistency analysis based on a voxel-wise correlation analysis between the maps (T-values) of both countries, with their slope analysis results for R (Rho = 0.12, *p* < .001) and for wSDM (Rho = 0.81, *p* < .001). All brain images are presented according to neurological convention.

1. **Supplementary figure 4:** SN consistency analysis without thresholding.


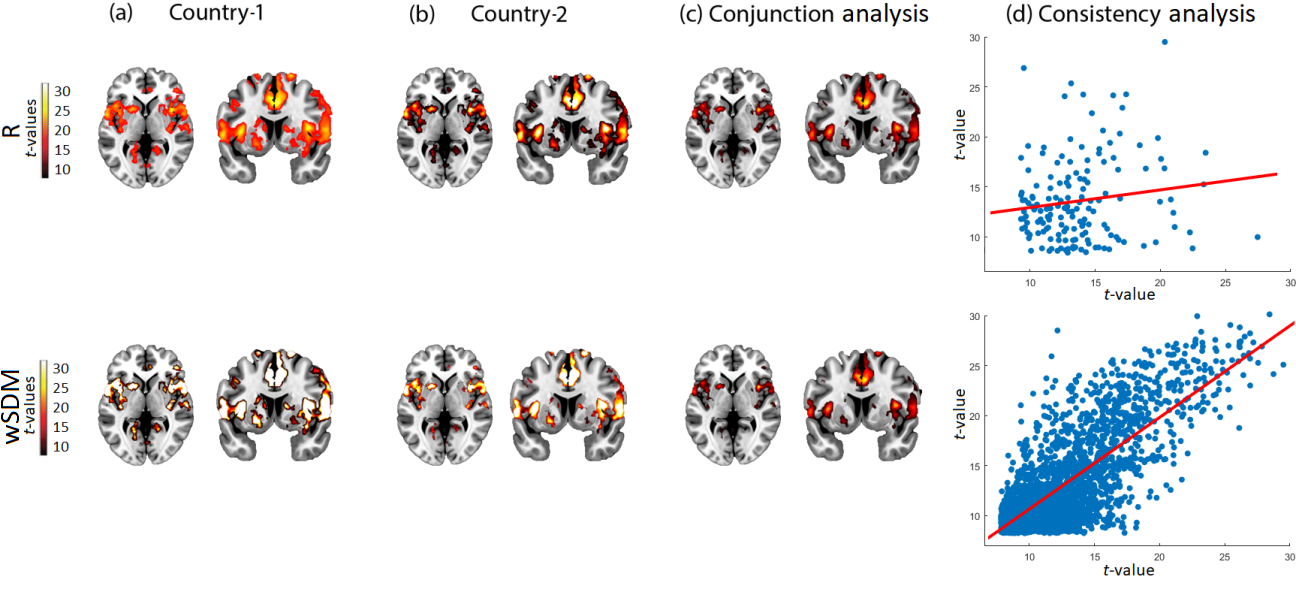


Supplementary Figure 4 SN consistency analysis without thresholding. (a)-(b) Seed analysis results of the SN using R and wSDM, for Country-1 and Country-2. (FWE-corrected, *p* = .05 on the voxel level, extent threshold = 30) (axial plane z = 3, coronal plane y = 6) (c) Cluster overlap between Country-1 and Country-2 (FWE-corrected, *p* = .05, extent threshold = 30 voxels). (d) Consistency analysis based on a voxel-wise correlation analysis between the maps (T-values) of both countries, with their slope analysis results for R (Rho = 0.31, *p* < .001) and for wSDM (Rho = 0.83, *p* < .001). All brain images are presented according to neurological convention.

1. **Supplementary figure 8:** Visual network.


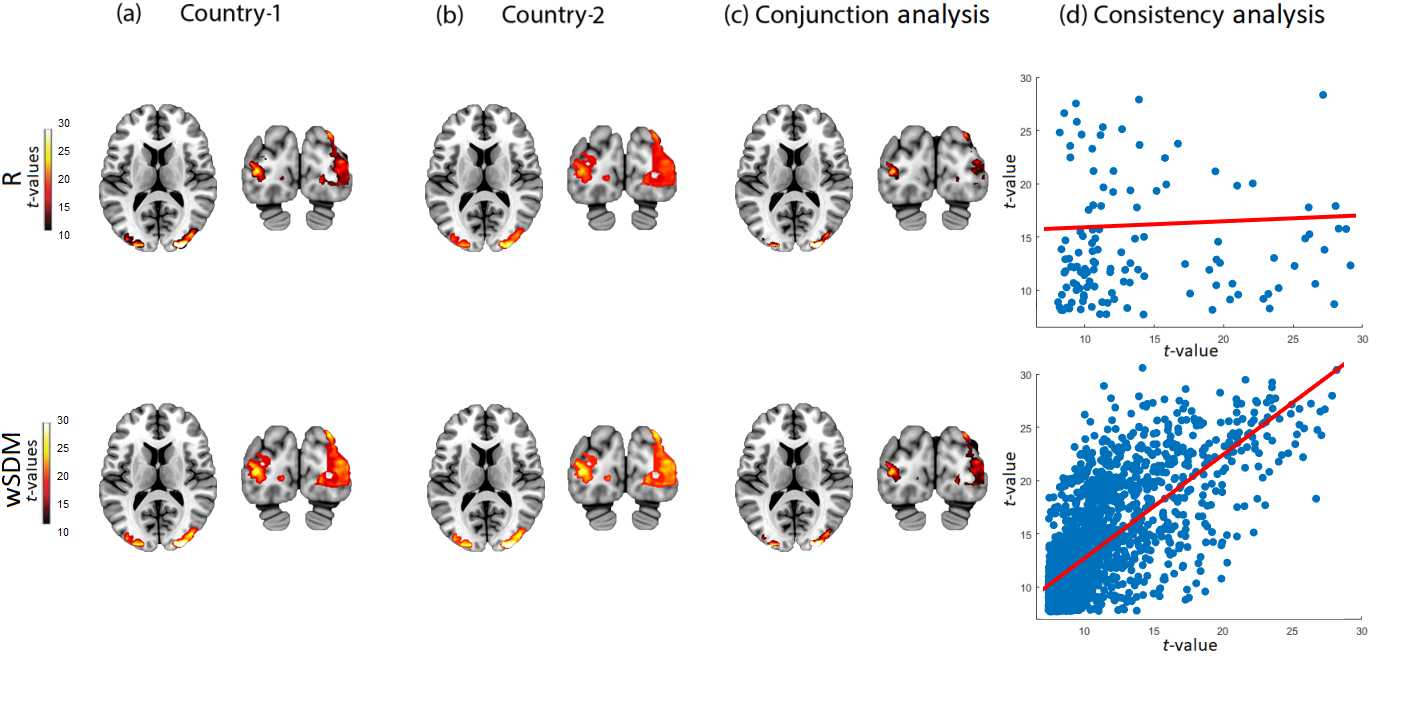


Supplementary figure 8. Visual network (SN). Seed analysis results of the primary visual cortex using R and wSDM at the 30th percentile threshold, (a-b) for Country-1 and Country-2 (FWE-corrected, *p* = .05 on the voxel level, extent threshold = 30). (axial plane z = 12, coronal plane x = -90). (c) Cluster overlap between Country-1 and Country-2 (FWE-corrected, *p* = .05, extent threshold = 30 voxels). (d) Consistency analysis based on a voxel-wise correlation analysis between the maps (*T*-values) of both countries, for R (Rho = 0.16, *p* < .001) while for wSDM (Rho = 0.86, p < .001). All brain images are presented according to neurological convention.

1. **Supplementary figure 9**: bvFTD vs. Controls FC in the visual network.


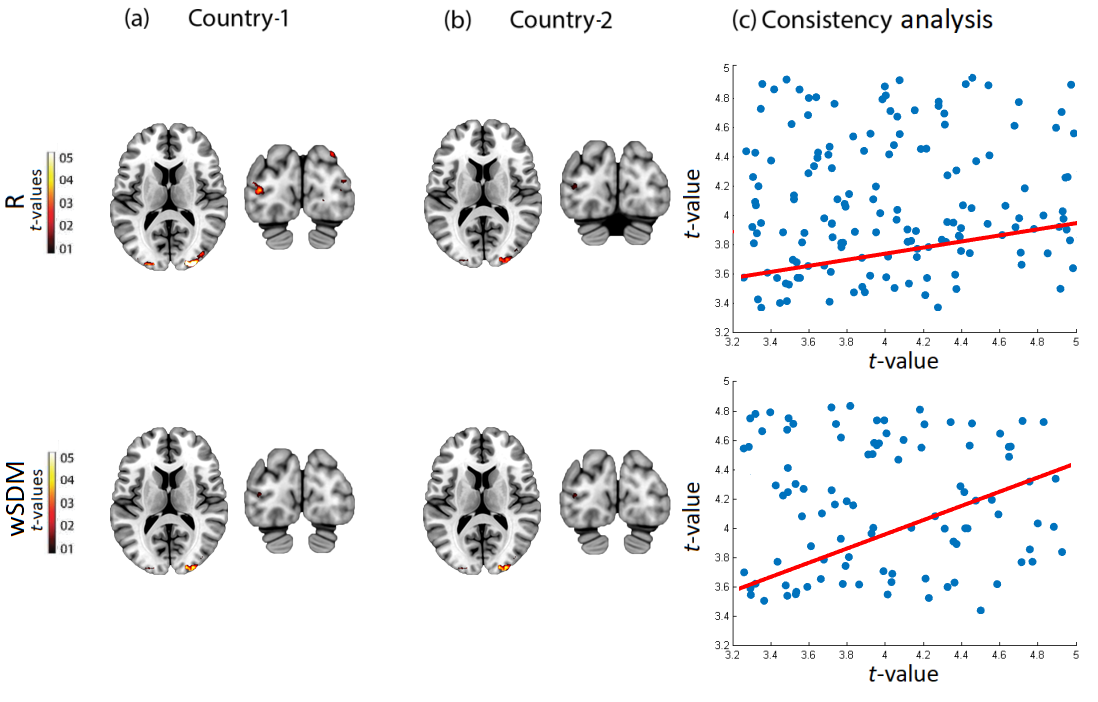


Supplementary figure 9. bvFTD vs. healthy controls. (a)-(b). Seed connectivity maps (axial plane z = 12, coronal plane y = -90) comparing HC > bvFTD for the primary visual cortex through a two-sample t-test. The connectivity maps were thresholded at the 30th percentile (*p* = .001 uncorrected, extent threshold = 30 voxels). (c) Consistency analysis based on a voxel-wise correlation analysis between the maps (T-values) of both countries based on a conjunction analysis, with their slope results for R (Rho = 0.31, *p* < .001) and for wSDM (Rho = 0.56, *p* < .001). All brain images are presented according to neurological convention.

1. **Supplementary figure 10**: Accuracy and ROC curves for Country-1 and Country-2, for R, DM, and wSDM (using the SVM classifier in the SN).


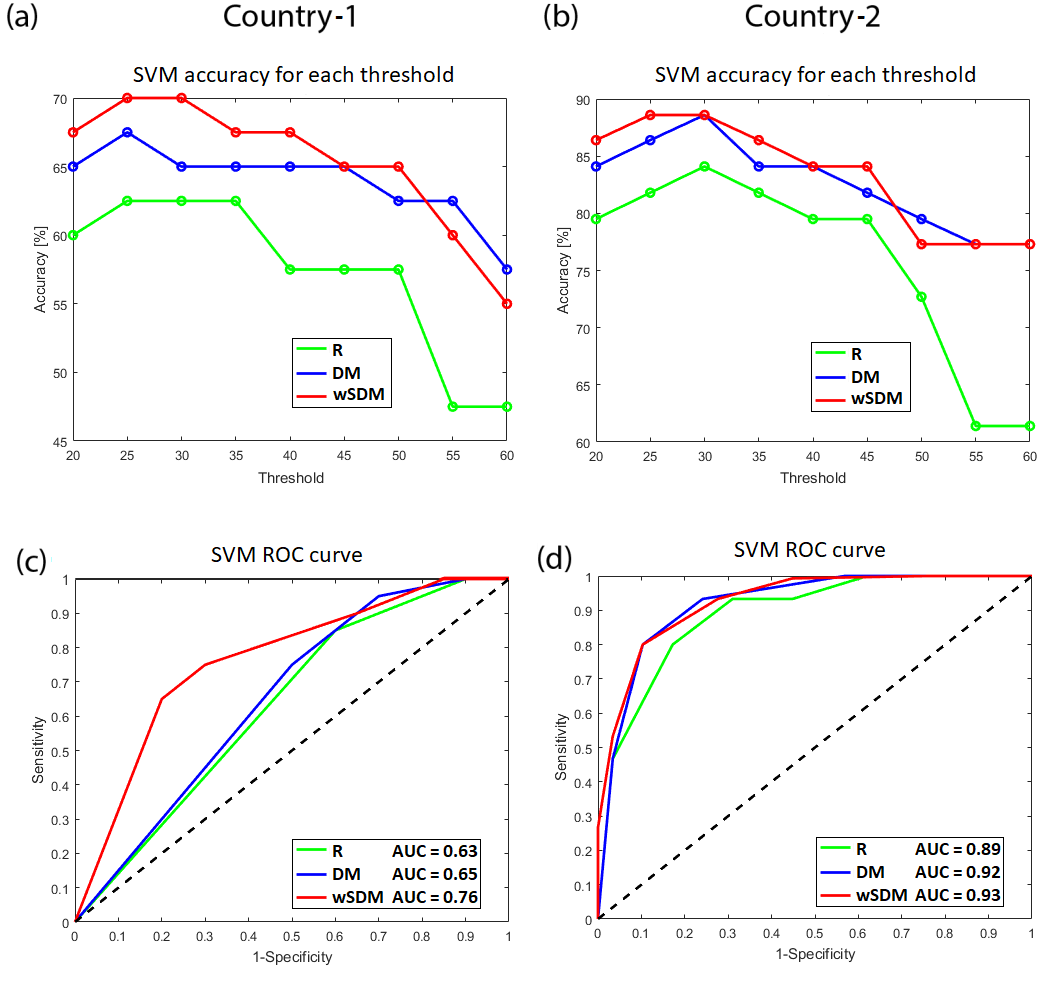


**Supplementary figure 10. Accuracy and ROC curves:** **(a) (b)** Classification accuracy rates under different thresholds and **(c) (d)** ROC curves (*p* < .01) at the optimal threshold for R, DM, and wSDM, for both countries.

1. **Supplementary figure 11:** Machine learning statistical Analysis for Country-1 and Country-2 (using the SVM classifier in the SN).


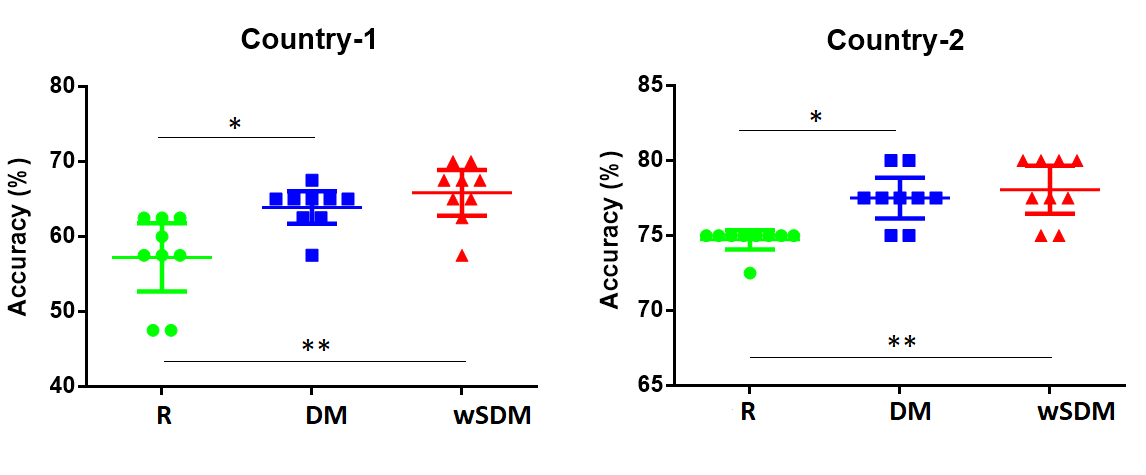


**Supplementary figure 11:** Means and 95% CI plot. **Wilcoxon test (Country-1):** * For R vs. DM: score = 72.5, *p* = .004. For DM vs. wSDM: score = 57.5, *p* = .14. ** For R vs. wSDM: score = 74, *p* = .0031. **Wilcoxon test (Country-2):**  For R vs. DM: score = 59.5, *p* = .0977. For DM vs. wSDM: score = 48.5, p = .501. * For R vs. wSDM: score = 63.5, *p* = .04.

1. **Supplementary figure 12:** Accuracy and ROC curves for Country-1 and Country-2, for R, DM, and wSDM (using the kNN classifier in the SN).


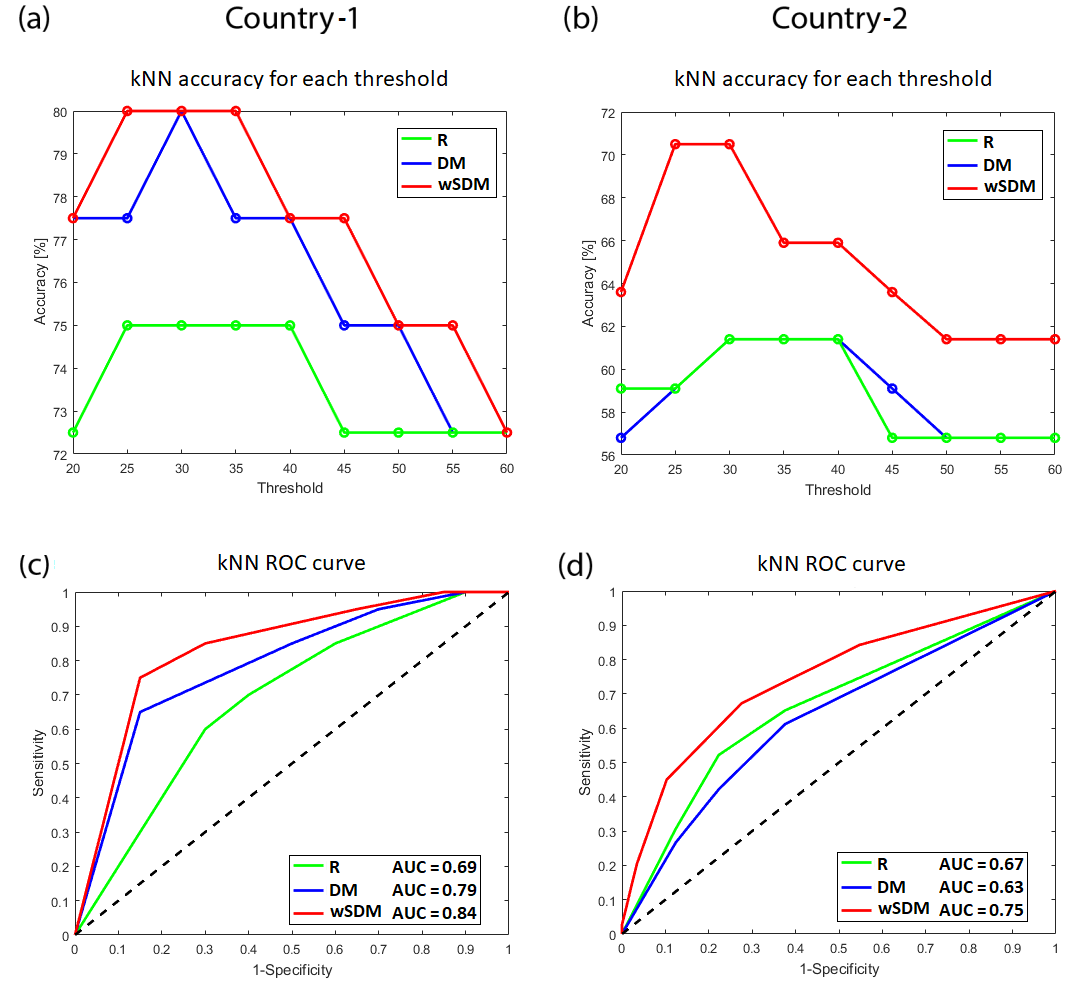


**Supplementary figure 12. Accuracy and ROC curves:** **(a) (b)** Classification accuracy rates under different thresholds and **(c) (d)** ROC curves (*p* < .01) at the optimal threshold for R, DM, and wSDM, for both countries.

1. **Supplementary figure 13:** Machine learning statistical analysis for Country-1 and Country-2. (using the kNN classifier in the SN).


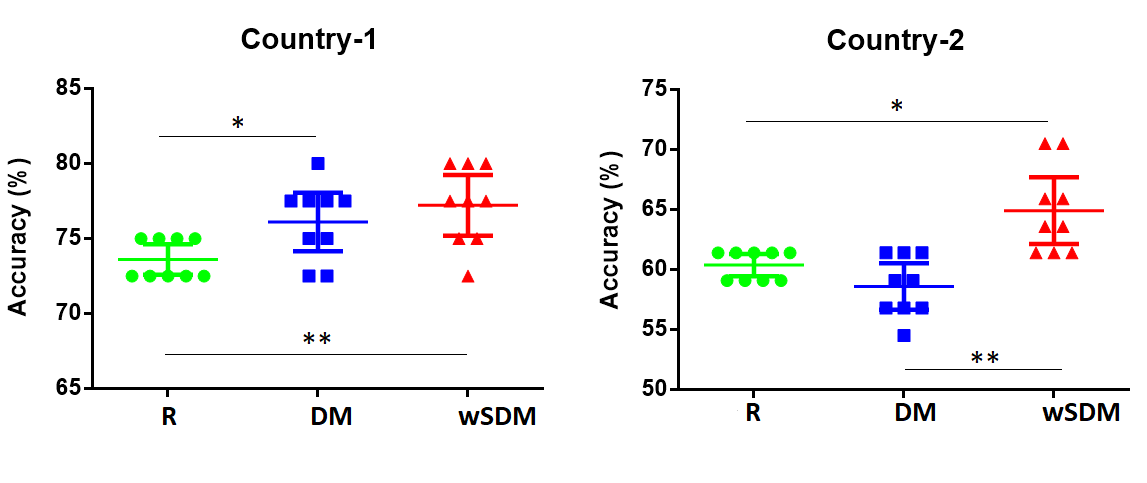


**Supplementary figure 13:** Mean and 95% CI plot. **Wilcoxon test (Country-1):** * For R vs. DM: score = 64, *p* = .03. For DM vs. wSDM: score = 50.5, *p* = .38. ** For R vs. wSDM: score = 70.5, *p* = .006. **Wilcoxon test (Country-2):**  For R vs. DM: score = 23.5, *p* = .11. * For R vs. wSDM: score = 73.5, *p* = .0024. ** For DM vs. wSDM: score = 76.5, *p* = .0013.

**References**

1 Farb, N. A. *et al.* Abnormal network connectivity in frontotemporal dementia: evidence for prefrontal isolation. *Cortex; a journal devoted to the study of the nervous system and behavior* **49**, 1856-1873, doi:10.1016/j.cortex.2012.09.008 (2013).

2 Garcia-Cordero, I. *et al.* Stroke and Neurodegeneration Induce Different Connectivity Aberrations in the Insula. *Stroke* **46**, 2673-2677, doi:10.1161/STROKEAHA.115.009598 (2015).

3 Sedeno, L. *et al.* Brain Network Organization and Social Executive Performance in Frontotemporal Dementia. *Journal of the International Neuropsychological Society : JINS* **22**, 250-262, doi:10.1017/S1355617715000703 (2016).

4 Sedeño, L. *et al.* Tackling variability: A multicenter study to provide a gold-standard network approach for frontotemporal dementia. *Hum Brain Mapp.* (2017).

5 Fox, M. D., Zhang, D., Snyder, A. Z. & Raichle, M. E. The global signal and observed anticorrelated resting state brain networks. *Journal of neurophysiology* **101**, 3270-3283, doi:10.1152/jn.90777.2008 (2009).

6 Murphy, K., Birn, R. M., Handwerker, D. A., Jones, T. B. & Bandettini, P. A. The impact of global signal regression on resting state correlations: are anti-correlated networks introduced? *Neuroimage* **44**, 893-905, doi:10.1016/j.neuroimage.2008.09.036 (2009).

7 Weissenbacher, A. *et al.* Correlations and anticorrelations in resting-state functional connectivity MRI: a quantitative comparison of preprocessing strategies. *Neuroimage* **47**, 1408-1416, doi:10.1016/j.neuroimage.2009.05.005 (2009).

8 Ashburner, J. & Friston, K. J. Nonlinear spatial normalization using basis functions. *Human brain mapping* **7**, 254-266 (1999).

9 Fox, M. *et al.* The human brain is intrinsically organized into dynamic, anticorrelated functional networks. *Proc Natl Acad Sci U S A.* **102**, 9673-9678 (2005).

10 Raichle, M. E. A paradigm shift in functional brain imaging. *The Journal of neuroscience : the official journal of the Society for Neuroscience* **29**, 12729-12734, doi:10.1523/JNEUROSCI.4366-09.2009 (2009).

11 Supekar, K. & Menon, V. Developmental maturation of dynamic causal control signals in higher-order cognition: a neurocognitive network model. *PLoS computational biology* **8**, e1002374, doi:10.1371/journal.pcbi.1002374 (2012).

12 Menon, V. & Uddin, L. Q. Saliency, switching, attention and control: a network model of insula function. *Brain structure & function* **214**, 655-667, doi:10.1007/s00429-010-0262-0 (2010).

13 Seeley, W. W. *et al.* Dissociable intrinsic connectivity networks for salience processing and executive control. *The Journal of neuroscience : the official journal of the Society for Neuroscience* **27**, 2349-2356, doi:10.1523/JNEUROSCI.5587-06.2007 (2007).

14 Uddin, L. Q., Kelly, A. M., Biswal, B. B., Castellanos, F. X. & Milham, M. P. Functional connectivity of default mode network components: correlation, anticorrelation, and causality. *Human brain mapping* **30**, 625-637, doi:10.1002/hbm.20531 (2009).

15 van den Heuvel, M. P. & Hulshoff Pol, H. E. Exploring the brain network: a review on resting-state fMRI functional connectivity. *European neuropsychopharmacology : the journal of the European College of Neuropsychopharmacology* **20**, 519-534, doi:10.1016/j.euroneuro.2010.03.008 (2010).

16 Agosta, F. *et al.* Brain network connectivity assessed using graph theory in frontotemporal dementia. *Neurology* **81**, 134-143, doi:10.1212/WNL.0b013e31829a33f8 (2013).

17 Pievani, M., de Haan, W., Wu, T., Seeley, W. W. & Frisoni, G. B. Functional network disruption in the degenerative dementias. *The Lancet. Neurology* **10**, 829-843, doi:10.1016/S1474-4422(11)70158-2 (2011).

18 Piguet, O., Hornberger, M., Mioshi, E. & Hodges, J. R. Behavioural-variant frontotemporal dementia: diagnosis, clinical staging, and management. *The Lancet. Neurology* **10**, 162-172, doi:10.1016/S1474-4422(10)70299-4 (2011).

19 Greicius, M. D., Srivastava, G., Reiss, A. L. & Menon, V. Default-mode network activity distinguishes Alzheimer's disease from healthy aging: evidence from functional MRI. *Proceedings of the National Academy of Sciences of the United States of America* **101**, 4637-4642, doi:10.1073/pnas.0308627101 (2004).

20 Zhou, J. *et al.* Divergent network connectivity changes in behavioural variant frontotemporal dementia and Alzheimer's disease. *Brain : a journal of neurology* **133**, 1352-1367, doi:10.1093/brain/awq075 (2010).

21 Pievani, M., Filippini, N., van den Heuvel, M. P., Cappa, S. F. & Frisoni, G. B. Brain connectivity in neurodegenerative diseases--from phenotype to proteinopathy. *Nature reviews. Neurology* **10**, 620-633, doi:10.1038/nrneurol.2014.178 (2014).

22 Koslov, K., Mendes, W. B., Pajtas, P. E. & Pizzagalli, D. A. Asymmetry in resting intracortical activity as a buffer to social threat. *Psychological science* **22**, 641-649, doi:10.1177/0956797611403156 (2011).

23 Shirer, W. R., Ryali, S., Rykhlevskaia, E., Menon, V. & Greicius, M. D. Decoding subject-driven cognitive states with whole-brain connectivity patterns. *Cerebral cortex* **22**, 158-165, doi:10.1093/cercor/bhr099 (2012).

24 Garcia-Cordero, I. *et al.* Feeling, learning from and being aware of inner states: interoceptive dimensions in neurodegeneration and stroke. *Philosophical transactions of the Royal Society of London. Series B, Biological sciences* **371**, doi:10.1098/rstb.2016.0006 (2016).

25 Van den Stock, J. & Kumfor, F. Behavioural variant frontotemporal dementia: At the interface of interoception, emotion and social cognition? *Cortex; a journal devoted to the study of the nervous system and behavior*, doi:10.1016/j.cortex.2017.08.013 (2017).

26 Uddin, L. Q., Supekar, K. S., Ryali, S. & Menon, V. Dynamic reconfiguration of structural and functional connectivity across core neurocognitive brain networks with development. *The Journal of neuroscience : the official journal of the Society for Neuroscience* **31**, 18578-18589, doi:10.1523/JNEUROSCI.4465-11.2011 (2011).

27 Lavagnino, L. *et al.* Reduced resting-state functional connectivity of the somatosensory cortex predicts psychopathological symptoms in women with bulimia nervosa. *Frontiers in behavioral neuroscience* **8**, 270, doi:10.3389/fnbeh.2014.00270 (2014).

28 Pannekoek, J. N. *et al.* Aberrant limbic and salience network resting-state functional connectivity in panic disorder without comorbidity. *Journal of affective disorders* **145**, 29-35, doi:10.1016/j.jad.2012.07.006 (2013).

29 Tu, P. C., Hsieh, J. C., Li, C. T., Bai, Y. M. & Su, T. P. Cortico-striatal disconnection within the cingulo-opercular network in schizophrenia revealed by intrinsic functional connectivity analysis: a resting fMRI study. *Neuroimage* **59**, 238-247, doi:10.1016/j.neuroimage.2011.07.086 (2012).

30 Wei, H. *et al.* Altered Effective Connectivity among Core Neurocognitive Networks in Idiopathic Generalized Epilepsy: An fMRI Evidence. *Frontiers in human neuroscience* **10**, 447, doi:10.3389/fnhum.2016.00447 (2016).

31 Church, J. A. *et al.* Control networks in paediatric Tourette syndrome show immature and anomalous patterns of functional connectivity. *Brain : a journal of neurology* **132**, 225-238, doi:10.1093/brain/awn223 (2009).

32 Brier, M. R. *et al.* Loss of intranetwork and internetwork resting state functional connections with Alzheimer's disease progression. *The Journal of neuroscience : the official journal of the Society for Neuroscience* **32**, 8890-8899, doi:10.1523/JNEUROSCI.5698-11.2012 (2012).

33 Dopper, E. G. *et al.* Structural and functional brain connectivity in presymptomatic familial frontotemporal dementia. *Neurology* **83**, 19-26 (2014).

34 Machulda, M. M. *et al.* Effect of APOE epsilon4 status on intrinsic network connectivity in cognitively normal elderly subjects. *Archives of neurology* **68**, 1131-1136, doi:10.1001/archneurol.2011.108 (2011).

35 Calhoun, V. D., Liu, J. & Adali, T. A review of group ICA for fMRI data and ICA for joint inference of imaging, genetic, and ERP data. *NeuroImage* **45**, S163-172, doi:10.1016/j.neuroimage.2008.10.057 (2009).

36 McKeown, M., Hansen, L. & Sejnowski, T. Independent component analysis of functional MRI: what is signal and what is noise? *Curr Opin Neurobiol.* **13**, 620–629 (2010).

37 Bandettini, P. A., Jesmanowicz, A., Wong, E. C. & Hyde, J. S. Processing strategies for time-course data sets in functional MRI of the human brain. *Magn Reson Med* **30**, 161-173 (1993).

38 Baudewig J, Dechent P, Merboldt KD & J., F. Thresholding in correlation analyses of magnetic resonance functional neuroimaging. *Magn Reson Imaging* **21**, 1121-1130, doi:10.1016/s0730-725x(03)00323-0 (2003).

39 Aurich, N. K., Alves Filho, J. O., Marques da Silva, A. M. & Franco, A. R. Evaluating the reliability of different preprocessing steps to estimate graph theoretical measures in resting state fMRI data. *Frontiers in neuroscience* **9**, 48, doi:10.3389/fnins.2015.00048 (2015).

40 Kundu, P. *et al.* Integrated strategy for improving functional connectivity mapping using multiecho fMRI. *Proceedings of the National Academy of Sciences of the United States of America* **110**, 16187-16192, doi:10.1073/pnas.1301725110 (2013).

41 Tomasi, D., Wang, R., Wang, G. J. & Volkow, N. D. Functional connectivity and brain activation: a synergistic approach. *Cerebral cortex* **24**, 2619-2629, doi:10.1093/cercor/bht119 (2014).

42 Pereira, F., Mitchell, T. & Botvinick, M. Machine learning classifiers and fMRI: a tutorial overview. *NeuroImage* **45**, S199-209, doi:10.1016/j.neuroimage.2008.11.007 (2009).

43 Cohen, J., Cohen, P., West, S. G. & Aiken, L. S. Applied Multiple Regression/Correlation Analysis for the Behavioral Sciences. *3rd Edn. Mahwah, NJ: Lawrence Erlbaum Associates, Inc.* (2003).

44 Zarogianni, E., Moorhead, T. W. & Lawrie, S. M. Towards the identification of imaging biomarkers in schizophrenia, using multivariate pattern classification at a single-subject level. *NeuroImage. Clinical* **3**, 279-289, doi:10.1016/j.nicl.2013.09.003 (2013).

45 Meier, T. B. *et al.* Support vector machine classification and characterization of age-related reorganization of functional brain networks. *NeuroImage* **60**, 601-613, doi:10.1016/j.neuroimage.2011.12.052 (2012).

46 Song, X. & Chen, N. K. A SVM-based quantitative fMRI method for resting-state functional network detection. *Magn Reson Imaging* **32**, 819-831, doi:10.1016/j.mri.2014.04.004 (2014).

47 Ghosh, A. K. On optimum choice of k in nearest neighbor classification. *Computational Statistics & Data Analysis* **50**, 3113-3123, doi:10.1016/j.csda.2005.06.007 (2006).

48 Wang, J. *et al.* Classification of fMRI patterns--a study of the language network segregation in pediatric localization related epilepsy. *Human brain mapping* **35**, 1446-1460, doi:10.1002/hbm.22265 (2014).

49 Oosterhof, N. N., Connolly, A. C. & Haxby, J. V. CoSMoMVPA: Multi-Modal Multivariate Pattern Analysis of Neuroimaging Data in Matlab/GNU Octave. *Frontiers in neuroinformatics* **10**, 27, doi:10.3389/fninf.2016.00027 (2016).

50 Shirer, W., Ryali, S., Rykhlevskaia, E., Menon, V. & Greicius, M. Decoding subject-driven cognitive states with whole-brain connectivity patterns. *Cereb Cortex.* **22**, 158-165 (2012).

51 Gaonkar, B. & Davatzikos, C. Analytic estimation of statistical significance maps for support vector machine based multi-variate image analysis and classification. *NeuroImage* **78**, 270-283, doi:10.1016/j.neuroimage.2013.03.066 (2013).
